# How effective is mHealth-supported home-based post-knee arthroplasty rehabilitation in improving knee function and continuum of care: protocol of an open label randomised controlled trial from India

**DOI:** 10.1136/bmjopen-2025-106469

**Published:** 2025-11-26

**Authors:** Siaa Girotra, Purnima Shrivastava, Ajit Kumar, Ruchika Madan, Seema Grover, Sahil Batra, Bhavuk Garg, Devarsetty Praveen, Susmita Chatterjee, Ankita Kasotia, Shyamashree Biswas, Manoj Soni, Sridevi Gara, Rajesh Malhotra, Ralph Maddison, Niveditha Devasenapathy

**Affiliations:** 1 The George Institute for Global Health, New Delhi, India; 2 Department of Orthopaedics, All India Institute of Medical Sciences, New Delhi, India; 3 Department of Geriatric Medicine, All India Institute of Medical Sciences, New Delhi, India; 4 Department of Physiotherapy and Rehabilitation, Indraprastha Apollo Hospitals, New Delhi, India; 5 The George Institute for Global Health India, New Delhi, India; 6 The George Institute for Global Health, Sydney, New South Wales, Australia; 7 Prasanna School of Public Health, Manipal, India; 8 Department of Orthopedics, Indraprastha Apollo Hospitals, New Delhi, India; 9 Deakin University Institute for Physical Activity and Nutrition, Geelong, Victoria, Australia

**Keywords:** Telemedicine, Mobile Applications, REHABILITATION MEDICINE, Clinical Trial, Knee

## Abstract

**Introduction:**

Monitoring physical rehabilitation is an essential component of patient recovery after knee arthroplasty. Monitoring can be remote, or clinic based. In India, unsupervised home-based physical rehabilitation is a common practice, but there is a lack of evidence to demonstrate the effectiveness of remote monitoring. Therefore, we developed and piloted a mobile application (TeleREhabilitation after knee ArThroplasty app) based on behaviour design thinking to support the recovery period. This trial aims to compare the effectiveness, acceptability, cost and safety of this app-supported home-based intervention against usual care using an open label, 1:1 individual randomised superiority trial at two tertiary care hospitals in India.

**Methods and analysis:**

Consecutive adults undergoing partial or total, unilateral or bilateral knee arthroplasty who can use a smart phone will be invited to participate in this trial. Consenting individuals will be randomised to either an app-supported intervention or a usual home-based rehabilitation which typically consists of provision of oral or written instructions at discharge and follow-up check-up with the surgeon or physiotherapist at their discretion or as per individual need. We aim to recruit 300 individuals over a period of eighteen months. The primary objective is to compare patient-reported knee function between the two groups at 3 and 6 months postsurgery. Secondary objectives are to compare patient-reported outcomes (pain and activity), performance-based outcomes (lower limb strength and knee function), resource utilisation and quality of life. Fidelity of implementation, end-user experiences and challenges in implementing this intervention will be measured using both quantitative and qualitative methods. Quantitative data will be analysed in Stata, and group comparisons will be done using mixed effect linear regression. A mixed-methods approach will be used to analyse and interpret the process evaluation data. A modified intention-to-treat approach will be taken, which includes all those who were randomised irrespective of their adherence to trial protocol if they had at least one follow-up visit after enrolment.

**Ethics and dissemination:**

The protocol has been approved by the ethics committees of the sponsor institute (The George Institute for Global Health) and the two clinical sites (All India Institute for Medical Sciences, Delhi & Indraprastha Apollo Hospitals, Delhi). The results will be disseminated via peer-reviewed publications, conference presentations and via plain language newsletters to the trial participants.

**Trial registration number:**

CTRI/2024/06/068838.

**STRENGTHS AND LIMITATIONS OF THIS STUDY**

- TeleREhabilitation after knee ArThroplasty trial is a randomised controlled trial to evaluate a multicomponent behavioural app-based intervention for monitoring rehabilitation among individuals following knee arthroplasty in India.
- The trial evaluates various clinical, patient-reported and cost outcomes at 3 and 6 months post arthroplasty.
- The study has an embedded process evaluation using mixed methods that will help understand the mechanistic pathway of intervention.
- The participants are aware of the group allocation; however, the outcomes are assessed by a blinded assessor.
- This trial may have limited generalisability as it is conducted only in two tertiary hospitals in India.

## Introduction

 Physical rehabilitation is essential in the first 3 months following knee arthroplasty for knee arthritis.^1^ The rehabilitation protocol generally includes pain management, muscle strengthening, restoration of knee range of motion (ROM), gait and balance training.^2^ This therapy is initiated before discharge from the hospital and gradually stepped up, according to the individual’s ability and desired level of knee function. This requires regular monitoring by a physiotherapist^2^ and the extent of this monitoring needs to be customised to individual needs.

The effectiveness of various methods of internet-based rehabilitation monitoring (ie, technologies that use internet for sharing information and communication), on knee pain and function, ROM, satisfaction and time to return to routine activities has shown mixed results.^3^ In this systematic review and meta-analysis,^3^ conducted to determine the effectiveness of internet-based telerehabilitation among patients after total joint arthroplasty (n=1020, 11 studies), no significant difference in outcomes of pain, ROM, function, satisfaction with rehabilitation outcomes/process or quality of life compared with face-to-face rehabilitation was found. Though the internet-based telerehabilitation showed better outcomes in physical functional tests, interpretation of the pooled results is challenging due to heterogeneity of interventions, context of the study and inconsistency in outcome measures. Additionally, a study aimed at exploring patients’ perceptions regarding telerehabilitation services received post total knee arthroplasty found telerehabilitation to be convenient^4^ and cost saving when compared with clinic visits and comparable in terms of safety.^5^ However, there is an absence in published work on use of technology for remote monitoring in India and other low-income and middle-income contexts as demonstrated by a recent scoping review.^6^

The usual rehabilitation care in India is mostly home-based with various levels of in-person supervision either at home or in clinic. Previous observational studies reported by our research group indicated a need for rehabilitation support to minimise out-of-pocket expenditure incurred due to home visits made by physiotherapists or clinic visits made by the patients.^7^ Specifically, patients found travel to hospitals and navigating within the hospital challenging, leading to poor adherence to rehabilitation protocols or receiving physiotherapy assistance from non-professional individuals.^7 8^ Moreover, healthcare providers also expressed a need for a technology that could help them to monitor progress with functional recovery and be able to modify the therapy plan.^9^

In response to this need for supervised home-based physical rehabilitation and a lack of available tools to support rehabilitation, we developed a multicomponent application (TeleREhabilitation after knee ArThroplasty (TReAT app)) to provide knowledge about recovery process, aid adherence to the rehabilitation therapy and to facilitate remote therapy planning.^9^ The intervention functions to address the challenges in rehabilitation were finalised using a behaviour design thinking approach in consultation with orthopaedic surgeons, physiotherapists and behavioural experts.^9^ The TReAT app is built on a platform (SMARThealth) which has been tested before in community health settings for clinical decision support of non-communicable diseases.^10^ To evaluate the effectiveness of this app-supported home-based rehabilitation and its potential for routine adoption in clinical care, we aim to test the effectiveness, acceptability and safety of this approach against usual home-based rehabilitation by means of a randomised controlled trial. We hypothesise that app-supported home-based rehabilitation will be superior to usual home-based rehabilitation with respect to knee function, knee pain and lower limb activity in patients undergoing arthroplasty for knee arthritis. Further, to understand if the proposed intervention leads to the expected behavioural change and subsequent changes in clinical outcomes, we will embed a process evaluation within this trial. This will also help us answer questions regarding generalisability and scalability of the intervention in varying contexts.

### Objectives

The trial objectives comprise effectiveness, safety, cost, intervention fidelity and usability measured using quantitative and qualitative research methods.

#### Primary objectives

To compare patient-reported knee function between app-supported home-based rehabilitation versus usual home-based rehabilitation at 3 and 6 months, in patients undergoing knee arthroplasty for knee arthritis.

#### Secondary objectives

The key secondary objectives are to compare the superiority of app-based rehabilitation versus usual home-based rehabilitation in patients undergoing arthroplasty for knee arthritis for knee pain, knee ROM, gait speed and stability, balance, lower limb strength and lower extremity activity at 3 and 6 months. Safety outcomes include falls and knee-related hospital readmissions.

#### Process evaluation objectives

We will embed a process evaluation informed by the theoretical framework proposed by Linnan and Steckler^11^ and the UK Medical Research Council process evaluation guidance for complex interventions,^12^ to understand the underlying mechanistic pathway of various components of the TReAT intervention and implementation of intervention in real world context. The specific objectives of the TReAT intervention process evaluation are to evaluate implementation fidelity, reach of the intervention, usage of the app, identify contextual barriers and facilitators for implementing the intervention and end-user satisfaction.

#### Cost evaluation objectives

We will assess the costs, effects (quality adjusted life-years), and cost–utility of this app-supported intervention against usual home-based rehabilitation.

## Methods and analysis

### Trial design

**Figure 1:**
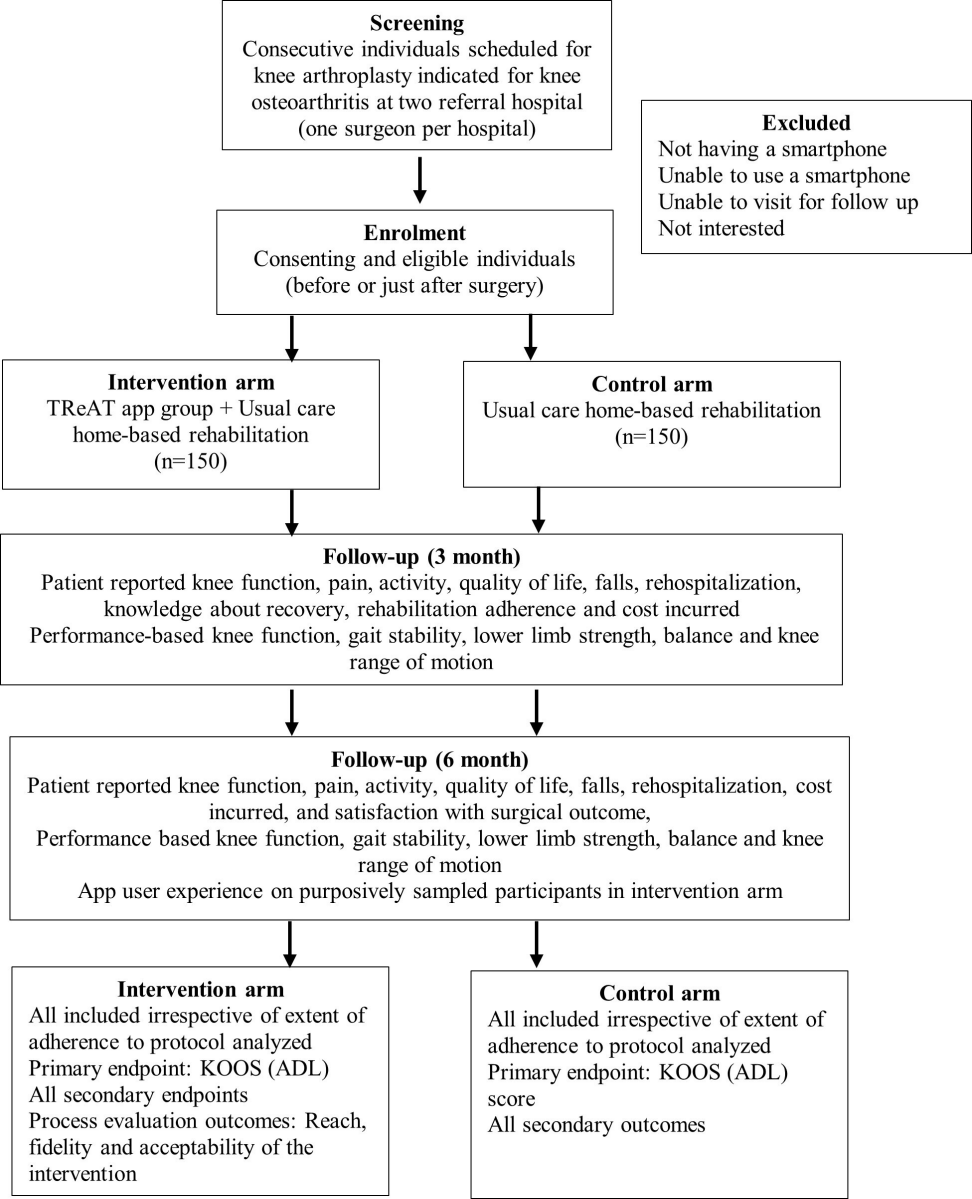
CONSORT trial flow. ADL, activities of daily living; CONSORT, Consolidated Standards of Reporting Trials; KOOS, Knee Injury and Osteoarthritis Outcome Score; TReAT, TeleREhabilitation after knee ArThroplasty.

This is a parallel arm 1:1 individual randomised open-label superiority trial with an embedded cost–utility analysis conducted at two tertiary care centres. Patients undergoing knee arthroplasty will be randomly allocated to app-supported or usual care home-based rehabilitation and followed up until 6 months after surgery (figure 1). The trial is registered with the clinical trial registry of India (CTRI/2024/06/068838). The trial recruitment commenced in June 2024 and is planned to finish in December 2025.

### Study setting

This trial will be conducted at a publicly funded and a privately funded tertiary care hospital in Delhi, India. In these hospitals, the usual approach for rehabilitation after knee arthroplasty is a home-based rehabilitation protocol with outpatient visits when required. Typically, discharge to home happens 48–72 hours after surgery and patients are given a printed pamphlet with details of the exercises to be performed postsurgery. Patients are free to seek external physiotherapy support. The study hospitals cater to a vast geographic area in the Northern region of India and a sociodemographically diverse population. A single operating unit at each of the hospitals will participate in this trial and they perform approximately 15–20 primary knee arthroplasties in a month.

### Eligibility criteria and recruitment

Consecutive patients scheduled for primary knee arthroplasty with the participating orthopaedic surgeon will be screened for eligibility. Patients will be screened at the outpatient orthopaedic surgery clinic or when admitted for the surgery or postsurgery but prior to hospital discharge.

#### Inclusion criteria

- Adults (aged ≥18 years) scheduled for primary, partial or total (unilateral or bilateral) knee arthroplasty indicated for knee osteoarthritis.
- Individuals with access to a smartphone and internet connection.
- Individuals, or any of the family members living in the same household, who can navigate a smartphone and use common features such as video calling, messaging and camera.
- Individuals able to speak and read English or Hindi language.

#### Exclusion criteria

- Individuals undergoing revision knee arthroplasty or knee arthroplasty indicated for trauma, tuberculosis of knee joint or other septic arthritis.
- Individuals unable to make a hospital visit at 3 and 6 months after surgery.
- Individuals with severe cognitive impairment precluding ability to provide fully informed consent.
- Individuals with uncorrectable hearing and visual impairment.
- Individuals not willing to be randomised to the rehabilitation monitoring approaches.

Eligibility will be assessed by the site investigator and referred to the research coordinator (with a background in physiotherapy) to explain the study procedures and obtain written consent for trial participation. Trial-related information in English or Hindi will be shared with the patient and the accompanying family member, using a printed document and an audiovisual aid. The information shared will include the purpose of research, method of allocation to two groups, participant rights, risk involved due to participation and to whom data about health and research data will be shared. Ample time and opportunities will be given to decide whether to participate, following which one of the site investigators or the research coordinator will obtain written informed consent. Reasons for ineligibility and non-participation will be documented.

### Interventions

#### Usual home-based rehabilitation

There are no standardised prehabilitation or postdischarge rehabilitation protocols followed in the participating hospitals. Hence, the comparator group will receive usual care, which typically consists of provision of oral or written instructions from the surgeon and physiotherapists at discharge. These instructions cover wound care, do’s and don’ts to prevent falls, and exercises to be followed until suture removal. Following suture removal, patients will attend a follow-up check-up with the surgeon, with or without a visit to the physiotherapy department. The frequency of visits will be at the surgeon’s discretion or as per individual need. Patients may also avail themselves of home-visit services by a physiotherapist external to the recruiting hospital.

#### App-based rehabilitation

**Figure 2:**
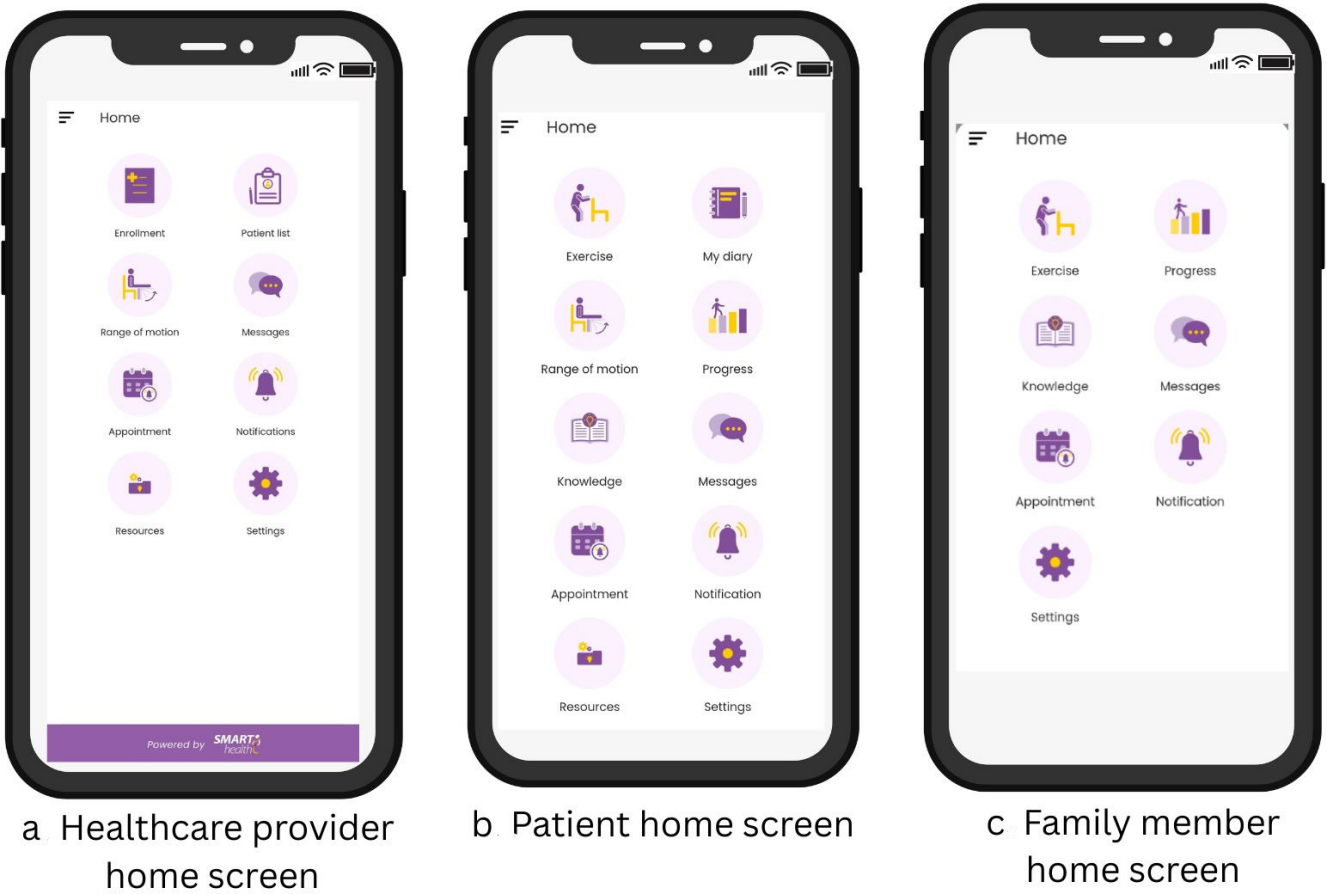
Snapshots of the TReAT application. TReAT, TeleREhabilitation after knee ArThroplasty.

The overarching goal of the intervention is to aid recovery, provide opportunities for the physicians to provide continuity of care and to reduce out-of-pocket expenses associated with the rehabilitation phase. The intervention has five interlinked components with defined functions and will be delivered via the TReAT app (figure 2, online supplemental efigure 1). The details of the stages of the intervention development have been published elsewhere. The intervention has the following components and functions:

1. Education to impart knowledge: A digital and a printed booklet with 12 chapters, consisting of context specific information related to preparing oneself for surgery, knowing about recovery process and important do’s and don’ts to safeguard the new knee, in English and Hindi will be made available at the time of enrolment (online supplemental efigure 2).

1. Training to skill them for doing the exercises: The users will have access to concise videos of exercises with English and Hindi voice-overs as an audio-visual aid. This will assist the patients to perform their exercises to improve ROM, muscle strength, balance and functional activities of daily living (ADL) (online supplemental efigure 3).
2. E-diary and goal setting to aid self-monitoring: Goal setting for the desired activity level will be discussed at the start of therapy. An e-diary will be available within the app to record pain, knee function, ROM, activity level and adherence to exercises, with an automatic graphical display of progress and congratulatory messages upon completion of exercise and activity goals. The family member will also be able to monitor the patient’s progress graphically. Push notifications will be sent periodically as reminders to perform physiotherapy exercises and complete the e-diary (online supplemental eFigure 4 and 5, eTable 1).
3. Opportunity to contact health care providers using e-connect: Two-way synchronous and asynchronous communication via audio, video and text features will be available (online supplemental eFigure 6).
4. Opportunity to e-monitor by healthcare provider: The app will facilitate continuity of care between healthcare provider and patients by allowing treatment plan updates based on the progress assessed through the progress graphs and remote consultations (online supplemental eFigure 5). The physiotherapists are provided with posters for each phase of the rehabilitation describing goals to achieve and progression criteria for the exercises. (online supplemental eFigure 7).

The comprehensibility of the education material, exercise videos, aesthetics and navigation of the app was assessed from 10 volunteering participants using the app prototype. The intervention was pilot tested on 30 participants and demonstrated high acceptability among users with digital literacy. Feedback from end users led to refinements in user interface, flexible scheduling of video consultations and addition of frequently asked questions and app manual. More details of the development of the app have been published previously.^9^ The duration of the proposed intervention is up to 6 months postsurgery. However, the dose of various components of the intervention will be dependent on each participant’s needs. We expect these components to achieve the proposed goal by improving their self-efficacy to follow the rehabilitation protocol (online supplemental eFigure 1). Periodic check-in calls will be made by the research coordinator to discuss and resolve any app usage queries. The research coordinator will also provide need-based support to the participating clinicians, for example, in enrolling participants into the app or reminding clinicians to schedule appointments. At the end of 6 months, participants will be unenrolled from the app. They will continue to have access to the education material in the app as long as they want. However, they will not be able to enter the e-diary and have a two-way communication with the healthcare providers. The only criteria to discontinue the app-based support before 6 months will be if the trial participant wishes to do so.

Participants in both groups will receive the usual presurgical and postsurgical care. The initiation of in-hospital rehabilitation, criteria for discharge and advice at the time of discharge will be similar for both groups. Clinic visits for suture removal or for any routine visit that the surgeon or physiotherapist may require will be on a need basis in both groups. The printed booklet will be given to participants enrolled in the usual care group during the 6-month trial visit. The dose, dosage and implementation of the intervention and usual care is summarised using the Template for Intervention Description and Replication^13^ (table 1).

**Table 1:** Overview of TReAT intervention in experimental and control groups according to the Template for Intervention Description and Replication checklist

TReAT intervention via mobile application	Usual home-based post-knee arthroplasty rehabilitation
Why?	
Poor adherence to physical rehabilitation after knee arthroplasty could lead to slow recovery of function. Lack of (opportunity) for continuum of care, self-management skills (capability) and self-efficacy (motivation) could be reasons for poor adherence. TReAT intervention was developed using behavioural design and design thinking approach, to deliver a multicomponent intervention package to improve patient recovery experience, adherence to exercise protocols and facilitate continuum of care following discharge to home.	There are no standardised rehabilitation monitoring protocols following discharge from hospital. The practices are varied within and across hospitals in India. The control group for this study is based on usual care.

What (materials) and Who?	
In addition to the usual care, the intervention has five interlinked components with a defined function delivered through the TReAT app. Digital and paper booklet in English and Hindi for education about recovery. Exercise videos as prescribed by healthcare provider with a voice-over in Hindi and English to aid exercise performance. Self-management and goal setting via e-diary to self-monitor progress (pain, range of motion, knee function, activity, and exercise adherence), reminders to complete exercises, congratulatory messages on completing goals. Sensitisation of family members to recovery needs via messages, access to patient progress graphs and education material. e-connect via two-way synchronous and asynchronous consultation via audio, video and text to promote communication. e-monitoring of progress by healthcare provider and modifying treatment plan using the e-repository of exercises.	Verbal rehabilitation instructions are provided by physiotherapists and surgeons at the hospital for pain management, wound care and exercises with or without written visual aids. Patients are expected to follow these instructions and are advised to visit clinics on a need basis. Face-to-face consultation in clinics is at a frequency determined by the clinical team.

What (procedures)?	
Physiotherapists and surgeons at the hospital will enrol, monitor and prescribe exercises via app. healthcare providers, patients and their caregivers will instal app from the application store (App store/Google Play Store) assisted by the research coordinator. A face-to-face demonstration of the app and provision of an app manual (English/Hindi) to patient and patients’ caregiver. Physiotherapists will be provided with therapy planning guidance posters. Before or after surgery during the hospital stay, physiotherapists will register the patient. Surgeon and physiotherapist will motivate the patient and caregiver to go through the education booklet. At the time of discharge physiotherapist will allocate exercises to be followed until the next review. Surgeon/physiotherapist will schedule the first video consultation at an agreed time, explain the importance of completing e-diary and motivate the patient/caregiver to go through the education booklet. Until 1 month after physiotherapist and surgeon will monitor patient progress daily, send personalised messages as required and request images/video of wound/functionality before the video consultation, and do the video consultation at a scheduled date and time. Physiotherapists will update the therapy plan, motivate patient depending on patients’ progress. Between 1 and 3 months physiotherapists will monitor patient weekly and follow-up as required. Post 3 months, the need for app-based support can be as per healthcare provider to a maximum of 6 months after enrolment.	Face-to-face consultation with surgeons and physiotherapists and exercise demonstrations at the respective clinics.

How and where?	
Healthcare providers, patients and their caregivers will have access to the app all the time through their mobile phone with good internet connectivity. Participants are expected to read the education materials, perform the exercises as per the therapy plan and complete pain and exercise daily and activity and knee function diary fortnightly. Healthcare providers can monitor patients’ progress remotely via progress graph, schedule one-to-one e-consultation for assessment and modify therapy plan based on pain, ability and current progress. For any concern, patients can message the healthcare provider and can request an e-appointment.	The patients can visit the hospital as and when their appointments are scheduled or appoint home services for physiotherapy support or visit private clinics. At the clinic, healthcare provider will complete a face-to-face physical assessment and modify the therapy as required.

When and how much?	
Intervention may start before or after surgery depending on the time of enrolment. Healthcare providers will provide support via app for a minimum of 3 months. The exercises and video consultation are tailored as per patient’s need with the rest of the intervention being constant. The patients will have access to the app as long as they want but the diary and messaging will stop until 6 months after surgery.	Individualised rehabilitation may start before or after surgery with varying duration and intensity. The number of visits to clinics depends on healthcare providers’ decision and patient preferences.

Tailoring	
Individualised exercises and e-consultation will be based on pain, functional progress and the patient’s expectation from the surgery. Messages will be tailored according to patients’ compliance of completing the diary and goals achieved.	Individualised exercises and consultation will be based on pain, ability, functional progress and patient’s expectation from the surgery.

How well (Fidelity)?	
A research coordinator will be available at clinical site to provide any technical assistance to the clinical team who will also have access to the dashboard to track the overall progress with the programme. Adherence to the intervention by patients will be assessed by the number of times they complete the diary, number of successful video consultations. Once a month check-in phone calls will be made to assist with any difficulty in using the app. Adherence to interventions by healthcare providers will be assessed by the number of scheduled video calls, and the number of times exercises have been updated.	There are no ways in usual care to monitor patient adherence to the rehabilitation protocol. However, to ensure trial participants are not lost to follow-up a monthly check-in call will be made to remind them about the follow-up visit.

TReAT, TeleREhabilitation after knee ArThroplasty.

### Assignment of interventions

Permuted varying block randomisation with 1:1 allocation ratio stratified by trial site was generated using Stata V.18 (StataCorp. 2023. Stata Statistical Software: Release 18., StataCorp) by an independent statistician, not involved in the recruitment process. Varying block size was used to minimise predictability due to the open-label nature of the trial. The randomisation scheme will be implemented using the interactive web randomisation feature of REDCap managed centrally at the sponsor institute. Following informed consent and baseline data collection, the research coordinator will log into REDCap using site specific user credentials to randomise the participant. The group allocation will be made available on confirmation of eligibility and informed consent. Automatic real-time system-generated notification of the group assignment will be sent to the trial investigators and research coordinator. All participants will be in the trial for 6 months after enrolment. They will be followed up at 3 and 6 months after randomisation.

### Blinding

This will be an open-label trial owing to the nature of the intervention. The outcome will be collected by a research coordinator who will be blinded to intervention assignment and will not be involved in recruitment and rehabilitation care of the participant.

### Outcomes

Primary outcome: Early recovery of knee function is one of the desired outcomes by patients undergoing knee arthroplasty.^14^ The primary outcome knee function will be assessed using the ADL subdomain of Knee Injury and Osteoarthritis Outcome Score (KOOS). It is a validated tool to evaluate post knee rehabilitation interventions^15^ and will be completed by the trial participants at baseline (before surgery status), 3 and 6 months after surgery in both groups. The total score ranges between 0 and 100 and the minimum clinically important group difference for this domain ranges from −1.5 to 15.5 units.^16^ The KOOS-ADL questionnaire will be self-administered in paper during the follow-up visit by the patient. If the patient is not able to complete it by self, then the family member will assist in eliciting the responses from the patient. In the absence of a family member, an independent assessor, not involved in the intervention implementation and rehabilitation, will administer the questionnaire. The primary comparisons would be to compare the end value of KOOS-ADL score at 3 and 6 months independently between the two groups adjusted for baseline score.

Secondary outcomes: Other quantitative outcomes include pain, assessed using the nine-item KOOS-Pain questionnaire^15^ with a 5-point ordinal response scale, higher scores indicating higher levels of pain. Activity level will be assessed using the lower extremity activity scale,^17^ a single item self-reported question with 18 states of activity, higher numbers indicating higher level of activity. Quality of life will be assessed using the EuroQoL-5 Dimensions-5 Levels questionnaire,^18^ capturing five domains of health, namely mobility, self-care, usual activities, pain/discomfort and anxiety/depression. The overall score ranges from 0 to 1, higher score indicating better quality of life. Safety, knowledge about recovery, rehabilitation adherence, satisfaction with surgical outcome and cost incurred will be collected using study-specific questionnaires. Active and passive knee ROM^19^ of the operated knee(s) will be measured using a digital goniometer. Performance-based measurements include chair rising test to assess lower limb strength, stair climbing and 6 min walk tests to assess knee function, timed-up and go to assess gait stability,^20^ and single leg stance to assess balance.^21^ Further explanation of the measurement tools is found in table 2 and the standardised procedure to conduct the performance-based tests is illustrated in online supplemental efigure 8.

**Table 2:** Trial outcomes and schedule of data collection

	Study period					
Time point	Enrolment	Baseline (0)		Visit 1 (3 months)		Visit 2 (6 months)
Screening	X		Telephonic health check-in		Telephonic health check-in	
Informed consent	X					
Health history (comorbidities)		X				
Randomisation	X					
Patient-reported outcome measures						
Knee function*		X		X		X
Knee pain†		X		X		X
Activity level‡		X		X		X
Quality of life§		X		X		X
Knee range of motion¶		X		X		X
Safety** (falls and rehospitalisation)				X		X
Knowledge about recovery††				X		
Rehabilitation adherence‡‡				X		
Cost incurred by patients§§				X		X
Satisfaction with surgical outcome¶¶						X
Performance based measures*** Function (SCT, 6MWT), Gait stability (TUG), Lower limb strength (CRT), Balance (SLS)				X		X
Process indicators††† Implementation fidelity and reach of the intervention, app usage, barriers and facilitators to implementation and end user satisfaction.	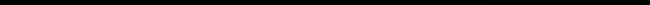					

* Knee function assessed using the Activities of Daily Living subdomain of KOOS, a self-reported 17-item questionnaire with 5-point ordinal scale responses, with score ranging from 0 to 100, higher score indicating better knee function.

† Knee pain will be assessed using the pain subdomain of KOOS (9 items) with 5-point ordinal scale responses. Score ranging from 0 to 100, higher score indicating severity of pain.

‡ Activity level assessed using lower extremity activity scale a single item question with 18 states of activity levels, higher level indicating higher activity.

§ Quality of life assessed using EQ-5D-5L, a 5-item questionnaire capturing 5 domains-mobility, self-care, usual activities, pain/discomfort, and anxiety/depression. The score ranges from 0 to 1, higher score indicating better quality of life. Additional Visual Analogue Scale for rating current state of health (0–100).

¶ Knee range of motion-active and passive flexion and extension of operated knee(s) using digital goniometer in supine position.

** Safety—number of falls after discharge to home as reported by the family or patient and number of rehospitalisations due to index knee surgery.

†† Knowledge about recovery-14 item studyp-specific questionnaire.

‡‡ Rehabilitation adherence-22-item study-specific questionnaire.

§§ Cost incurred by the patient after surgery for physiotherapy services and medications, travel, re-hospitalisation and potential productivity loss.

¶¶ Satisfaction with surgical outcome—8-item Likert scale questionnaire.

*** Performance based measures—SCT measured as time in seconds to complete climbing up and down 12 steps of 18 cm in vertical height; 6 minute walk testT-Distance covered in 6 min at self-paced walking; TUG- Time (in secs) taken to rise from a chair of 44 cm height, walk for 3 m, turn back and be seated on the same chair. CRT-number of repetitions in 30 s to raise rising chair of 44 cm height; SLS—time in seconds an individual can stand on a single leg with eyes open.

††† Process indicators will be assessed by triangulating the findings from the screening logs, research coordinator observations, app analytics data and qualitative interviews with the end users.

CRT, chair rising test; EQ-5D-5L, EuroQol-5 Dimensions-5 Levels; KOOS, Knee Injury and Osteoarthritis Outcome Score; 6MWT, 6 Minute Walk Test; SCT, stair climbing test; SLS, single leg stance; TUG, Timed Up and Go.

Process evaluation outcomes: Fidelity of the intervention and app usage by the patient and healthcare provider will be documented using a checklist. The data for this checklist will be extracted from the research coordinators’ notes and backend app analytics on app usage, by a member of the research team not involved in participant recruitment and follow-up. The reach of the intervention will be assessed from the screening logs. Contextual factors that act as facilitators and barriers for implementing this intervention and acceptability of the intervention will be collected through a semistructured interview with the end-users. To gain understanding of the mechanistic pathway to change, we will measure self-efficacy to rehabilitation. This questionnaire was developed based on the rehabilitation adherence questions designed for sports-related knee injury by Shin *et al*.^22^ However, our questionnaire is not validated for all the psychometric properties. We will also record knowledge on recovery after surgery using a set of knowledge questions and collect the list of exercises performed from both the groups.

Economic component: Resource use from patient perspective that includes cost of physiotherapy services, travel expenditures and wages lost due to clinic visits will be measured using study-specific questionnaires at 3 and 6 months in both groups. Health-related quality of life will be measured using EQ-5D-5L^18^ to measure quality-adjusted life-years (QALYs).

### Sample size

The primary endpoints are group differences in self-reported knee function measured using the KOOS-ADL at 3 and 6 months using mixed-effect linear regression. The KOOS-ADL score ranges from 0 to 100 with higher scores indicating better function. We aim to detect a difference of at least 7 points,^16^ and a common SD of 15 points was assumed. The sample size was estimated assuming two-sided, two-sample equal-variance t-test, with an overall type I error rate (α) of 0.05. The effect size is defined as d=(μ1−μ2)/σ, where σ is the common SD for both groups. To detect a population effect size of 0.40 with 85% marginal power, the number of needed participants will be around 150 in each group after adjusting for 10% loss to follow-up and multiplicity due to two primary time points using Bonferroni’s correction. The sample size was computed using PASS 2023, V.23.0.2. We expect to reach the target sample size in 18 months.

### Data collection and management

Data will be collected at the physiotherapy clinic of the recruiting hospitals in paper forms at baseline and at 3 and 6 months postrandomisation by a trained research coordinator. Data will be single entered into a web-based electronic data capture system (REDCap) with in-built validation checks and a query management system. Source data verification will be done by the trial manager to check for accuracy against the paper forms. Initially, 100% of forms will be checked and the subsequent percentage of the forms checked will depend on the proportion of transcription errors identified. Data related to cost will be entered into an excel sheet for further processing.

To ensure data quality, periodic data review checks will be done by the trial manager not involved in data collection, using Stata to flag discrepancies that may not be detectable by inbuilt automatic checks. These checks include cross-form logic checks and completeness of critical fields. Queries will be raised in REDCap for the research coordinator to update or respond. A back-up of all data will be done periodically as per the institution server policy. The paper forms, along with informed consent, will be safely secured in a cabinet with restricted access at the Sponsor institute.

Semistructured interviews will be conducted either face-to-face or telephonically from a purposively selected sample of trial participants based on the extent of app usage (users, partial-users, non-users). The interviews will be audio-recorded or written notes will be taken depending on how comfortable the participants are with the audio recording. Participants will be invited for interviews until data saturation is reached. All the healthcare providers involved in the intervention implementation at the hospital will be interviewed. The interviews will be conducted by a member of the research team trained in qualitative interview methods. Qualitative interviews will be transcribed and translated by one of the members of the research team and cross-checked by another member of the team for correctness and appropriateness.

### Participant retention strategies

The research coordinator involved in recruitment will telephonically contact the trial participants of both groups at least once a month for rapport building and to enquire about their health. For the app group participants, any technical query related to the app will also be resolved. Travel reimbursements will be provided to trial participants at 3-month and 6-month visits, as per the clinical site ethics committee’s recommendation. We will share a newsletter in English and Hindi every 2 months with all trial participants to provide an update on trial progress and encourage them to visit the hospital for their trial related follow-up. If a participant is unable to make it to the hospital for a follow-up visit, participant reported outcome measures will be collected telephonically and the participant will be motivated to visit the hospital at the earliest for conducting the performance-based measurements.

### Data confidentiality

Data entered in REDCap will not capture any personal identification and will be known by a unique identifier. Only the recruiting healthcare provider and the research coordinator employed for the trial will have contact details of the trial participant and the immediate family member to facilitate their follow-up. The name of the trial participant and phone number will be visible in the app that will be accessible only to healthcare providers. However, when the data are extracted from the app for fidelity analysis, the identifiers will not be extractable by the trial manager. The data contained in the app will be stored in a high security server accessible only to designated technical team leaders of the sponsors via a dedicated wide area network service. The server undergoes daily back-ups, with daily snapshot recovery points and 30 days’ retention. Anonymous participant data will be stored in REDCap and archived as per institutional archival policy. Qualitative data will be stored securely with restricted access on the sponsor server. The data in the app platform will be destroyed 5 years after completion of the trial.

### Patient and public involvement

During the development of the TReAT intervention, patients who had undergone knee arthroplasty and their family members were involved in pilot testing the different components of the TReAT app—the education material, exercise videos and diary questions. Patients were not involved in deciding on the choice of outcome, trial follow-up or any aspects of conduct of the study. However, during the conduct of this trial, bimonthly newsletters will be shared with all trial participants to provide an update on trial progress. Stakeholder consultations will be held to share findings and gain end user feedback on the usability and implementation of the TReAT app. A lay summary using infographics will be shared with trial participants via a newsletter at the end of the study.

### Statistical methods

#### Statistical methods for primary and secondary outcomes

Effectiveness outcomes: Baseline characteristics including mean, SD, number and percentage will be described. Mean group differences and 95% CI of KOOS-ADL score at 3 and 6 months adjusted for baseline score will be calculated using mixed effect linear regression. A p value of 0.025 will be considered as statistically significant for each of the time points. The analytical approach will be modified intention-to-treat, which includes all those who were randomised irrespective of their adherence to trial protocol if they had at least one follow-up visit after enrolment. This approach will exclude those who were lost to follow-up after enrolment.

All secondary continuous outcomes that include KOOS-Pain, ROM and performance-based measures will be compared at 3 and 6 months using linear mixed effect method adjusted for baseline KOOS-ADL, as performance-based measures will not be collected at baseline. A statistical analysis plan will be developed before the end of trial recruitment and made publicly available before final analysis. No interim analysis will be performed. The preplanned subgroups are gender and laterality of index surgery (bilateral/unilateral). Effect modification will be claimed based on interaction p<0.05. We will not perform a per-protocol analysis. Multiple imputation of missing primary outcome will be done only if more than 5% of enrolled are lost to follow-up. All analyses will be done in Stata (StataCorp) and R version 4.4.1 or higher (Postit Team, 2025).

Process evaluation: App usage (usage of diary, type of exercise prescribed and number of times therapy was modified) will be presented graphically along with suitable summary measures. The written research coordinators’ notes will be quantified using a fidelity checklist. Transcripts of qualitative interviews will be coded independently in NVivo software (Lumivero (2023) NVivo (V.14)), using an inductive-deductive approach followed by thematic analysis. The qualitative findings from the interviews, fidelity checklist, rehabilitation adherence and knowledge score, and functional outcomes will be triangulated to understand the mechanistic pathway to change, whether intervention use/non-use led to different outcomes. Further, contextual factors affecting the implementation and usage of the intervention will be summarised.

Cost analysis: Costing components will be considered from the patients’ perspective in both groups. We will assume equivalent costs for surgery, surgery-related in-hospital stays and medication in both groups and hence, costs for these components will not be collected. However, costs associated with any re-hospitalisation following discharge, either related to index surgery or other medical reasons, will be included in the analysis. Only patients who undergo unilateral or bilateral knee arthroplasty will be included in the cost analysis, and patients who will undergo knee arthroplasty of the other knee during the trial period will be excluded.

Responses to EQ-5D-5L will be converted to a single-index utility score using the Indian value set and converted into QALYs.^23^ QALY is based on the single utility score which defines the valuation of health-related quality of life from a patient’s perspective on a scale indicating 0 as ‘dead’ and 1 as ‘perfect health’. Assuming a linear trend in a patient’s utility scores from the baseline to each follow-up time point, the area under the resulting curves indicates the QALYs experienced by that patient at 6 months.^24 25^

The average of resource use, costs and EQ-5D-5L utility scores for both trial groups will be reported along with suitable method of dispersion. Comparison of cost and utility scores between two groups will be reported as mean difference and 95% CI. Bootstrapping methods will be used to address the expected skewed nature of these variables. We will report the incremental cost-effectiveness ratio representing the difference in cost for an additional QALY gained and cost per 5° knee flexion gained in the intervention group compared with usual care. Time-saving potential of the app-supported intervention as compared with the usual care will also be compared.

### Trial management

This is an investigator-initiated project with a low-risk intervention and no anticipated potential harm to participants, thus the trial will be overseen by the project coordination team with the principal investigator taking up the responsibility of trial oversight. Hence, a data safety monitoring committee was not appointed for this trial. The project coordination team consists of the trial manager, research coordinators and the principal investigator to provide oversight of the day-to-day management of the trial, review trial progress with recruitment, retention and data quality. Any protocol amendments related to patient eligibility criteria, increase in sample size, duration of trial participation and change in prespecified trial outcomes will be approved by the ethics committees before implementation and updated in the trial registry.

## Ethics and dissemination

The trial will be conducted in accordance with the principles of Declaration of Helsinki and Good Clinical Practice. The protocol was approved by the independent ethics committee at The George Institute for Global Health, India (32/2023), All India Institute of Medical Sciences, Delhi (AIIMSA00323/12.01.2024, RP-4/2024) and Indraprastha Apollo Hospitals, Delhi (IAH-BMR-004/01-24). Written informed consent will be received from the participants before enrolment in the study. After trial publication, the trial results will be published in a peer-reviewed biomedical journal irrespective of the direction of trial findings. The trial protocol, experiences of conducting this trial and trial results will be presented in national and international rehabilitation and physiotherapy-related conferences. A lay summary using infographics will be shared with trial participants via a newsletter at the end of the study. Stakeholder consultations will be held with volunteering trial participants, patients, healthcare providers, hospital administration, mHealth experts and app technology team to share findings, identify areas of improvement in the intervention package and discuss the scalability of this approach in routine care.

## Discussion

Personalised support during the rehabilitation period following knee arthroplasty, either in person or remotely, is shown to be beneficial when compared with unsupervised home-based rehabilitation.^26^ Studies from high-income countries have shown short-term benefit in some functional outcomes, safety and acceptability of use of technology for remote rehabilitation and monitoring.^5^ Hence, the use of technology to enable remote monitoring can improve access to healthcare professionals, especially to the elderly population who are dependent on family members to visit the hospital or physiotherapy clinic.^8^

India is making progress in telehealth initiatives^27^; however, locally developed technological innovations for remote monitoring after knee arthroplasty with end user involvement are a novel^6 9^ initiative. The TReAT app is built on the SMARThealth platform that holds the potential to be easily expanded to other areas of musculoskeletal rehabilitation such as ankle or shoulder injuries. The inclusion of a family member interface is unique to the TReAT intervention and will be very relevant to the Indian context. This could be attributed to widespread familial involvement in rehabilitation care and postoperative monitoring in India.

The success of this complex intervention will depend on several factors related to the intervention. First, familiarity and comfort of the healthcare providers and patients in using the TReAT app. Second, the additional steps that the intervention adds to the usual clinical workflow such as demonstration of the TReAT app to patients and family members, registering the patient into the app, assigning and updating therapy, fixing appointments after viewing patient progress and regularly communicating with the patients through messaging. Third, the intervention components such as reading the education material, completing the diary, viewing the exercises, being adherent to the tele/video appointments are components requiring active participation by the patients. Finally, the pathway to change to intended outcome on functional outcomes is dependent on a series of behaviour changes. We assume that the provision of reliable education improves patients’ awareness on pain management and helps in goal setting. Personalised therapy videos would help in achieving better exercise adherence leading to improvement in ROM and muscle strength.

**Figure 3:**
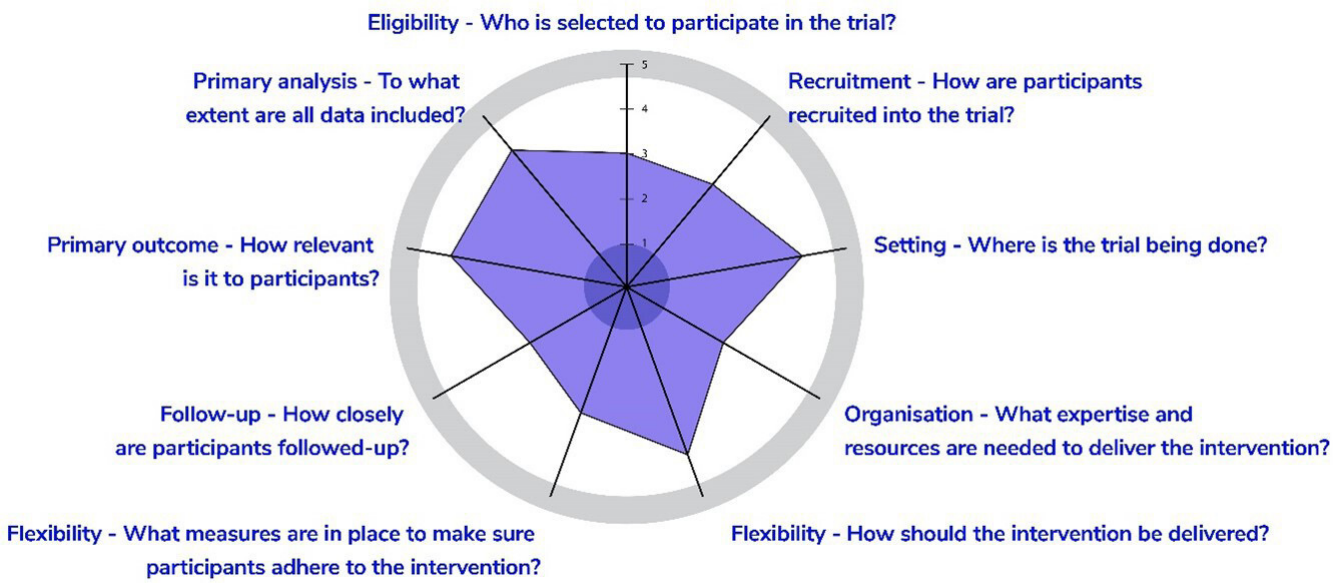
PRagmatic Explanatory Continuum Indicator Summary 2 wheel to demonstrate level of pragmatism of TReAT trial. Eligibility, 3: Eligibility limited to digitally literate individuals, thus limiting the applicability of the results about remote rehabilitation to patients or family members without a device or digital literacy. Recruitment path, 3: Recruiting only eligible patients operated by principal investigator at each site, instead of all patients referred to physiotherapy for post-knee replacement rehabilitation care. Setting, 4: Though only 2 participating referral hospitals across India, they cater to a large representative population in Northern India across socioeconomic status. Organisation intervention, 2.5: Minimal training provided to health care providers but requiring research coordinator support to enrol participants in the application and facilitate follow-up. Flexibility of intervention delivery, 4: The intervention components are delivered as per participant requirement; there is no restriction on concomitant therapies. Flexibility of intervention adherence, 3: In addition to usual care, the research coordinator makes check-in calls to all trial participants to aid on app use and reminders are given to health care providers for video consultation and therapy planning. Follow-up, 2.5: Patients are required to come for trial-related follow-up visits at 3 and 6 months postsurgery which is not part of usual care. Additionally, telephonic check-in calls made between visits to reduce attrition are additional to the trial. Outcome, 4: Patient-reported function is the primary outcome, but is not recorded as part of routine medical records and collected specifically for the trial. Analysis, 4: Modified intention-to-treat approach will be used to account for attrition. TReAT, TeleREhabilitation after knee ArThroplasty.

The TReAT trial, which will be conducted across two clinical sites, has been designed to be an explanatory trial with certain pragmatic elements such as flexibility of intervention delivery as per the requirements of the participants and the use of a patient-reported function as the primary outcome (figure 3). On completion, this trial will provide valuable evidence for acceptability of this approach and data to inform further technological refinements. Further, our process evaluation will also help to interpret findings and improve future iterations and understand potential implementation issues. If accepted in routine care, especially in public-funded hospitals, it could expand access to the quality of postdischarge care in remote regions of the country.

### Trial status

At the time of writing the manuscript, the trial is ongoing. Trial recruitment commenced in June 2024 (Study protocol version 1.0 dated third November 2023) and is planned to finish in December 2025. Data collection is planned to continue until June 2026.

## Supplementary material

10.1136/bmjopen-2025-106469 online supplemental file 1
