# Supplementary material for: How effective is mHealth-supported home-based post-knee arthroplasty rehabilitation in improving knee function and continuum of care: protocol of an open label randomised controlled trial from India
Source: BMJ Open. 2025 Nov 26;15(11):e106469. doi: 10.1136/bmjopen-2025-106469 (PMC12658543; doi:10.1136/bmjopen-2025-106469)

**Supplementary file**

**How effective is mHealth supported home-based post knee arthroplasty rehabilitation in improving knee function and continuum of care: protocol of an open label randomized controlled trial from India**

Authors: Siaa Girotra, Purnima Shrivastava, Ajit Kumar, Ruchika Madan, Seema Grover, Sahil Batra, Bhavuk Garg, Devarsetty Praveen, Susmita Chatterjee, Ankita Kasotia, Shyamashree Biswas, Manoj Soni, Sridevi Gara, Rajesh Malhotra, Ralph Maddison, Niveditha Devasenapathy

**List of Figures and tables**

[eFigure 1 Interacting components of TReAT intervention 2](#_Toc210394752)

[eFigure 2 Contents of the education material 3](#_Toc210394753)

[eFigure 3 Repository of exercise videos for therapy planning 4](#_Toc210394754)

[eFigure 4 My diary functionality 5](#_Toc210394755)

[eFigure 5 Progress graphs for monitoring 6](#_Toc210394756)

[eFigure 6 Messaging and video consultation functionality 10](#_Toc210394757)

[eFigure 7 Therapy planning posters 11](#_Toc210394758)

[eFigure 8 Standardised instructions for conducting performance-based functional tests 17](#_Toc210394759)

[eTable 1 Motivational messages (Participant) 7](#_Toc210039200)

In this supplementary document we describe the details of the TReAT intervention that aims to provide rehabilitation support to patients after knee arthroplasty and help healthcare providers with continuum of care. The intervention has five interlinked components- education, exercise training, goal setting and self-monitoring, communication and remote monitoring. We envision the intervention components to work in synchrony and provide knowledge, motivation and support to the patients throughout their rehabilitation period leading to better functional outcomes.

**
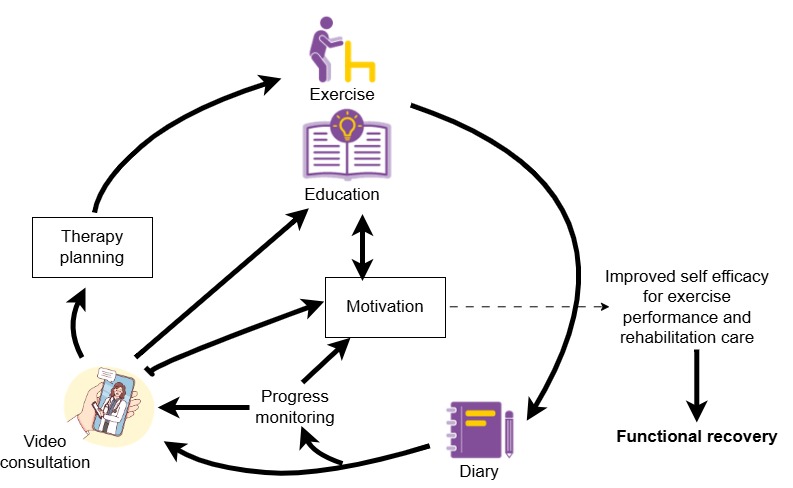
**eFigure 1 Interacting components of TReAT intervention


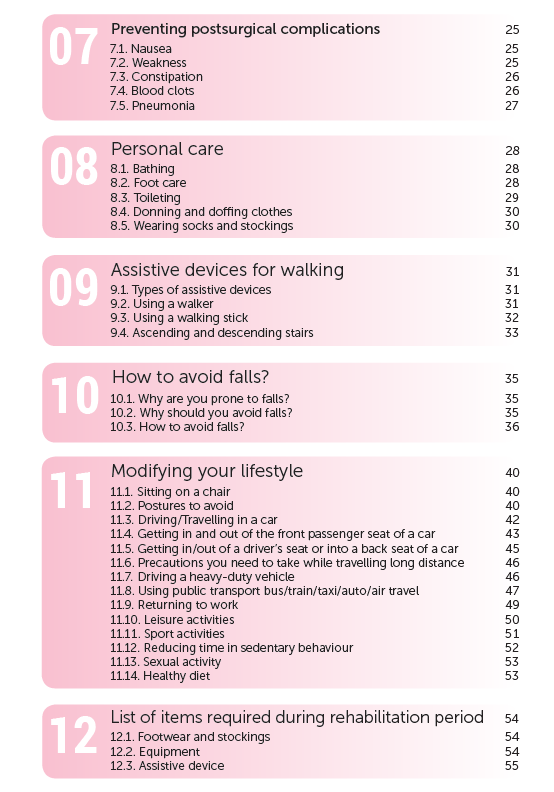


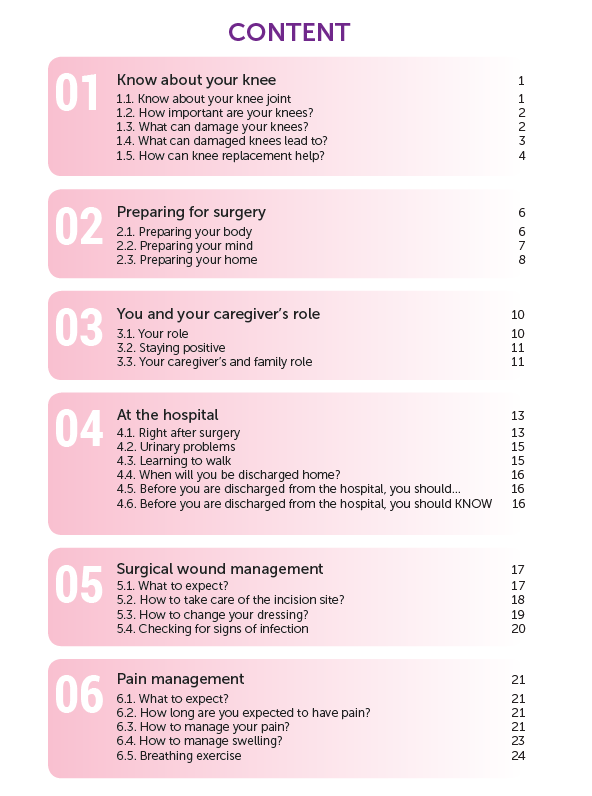
eFigure 2 Contents of the education material

eFigure 3 Repository of exercise videos for therapy planning
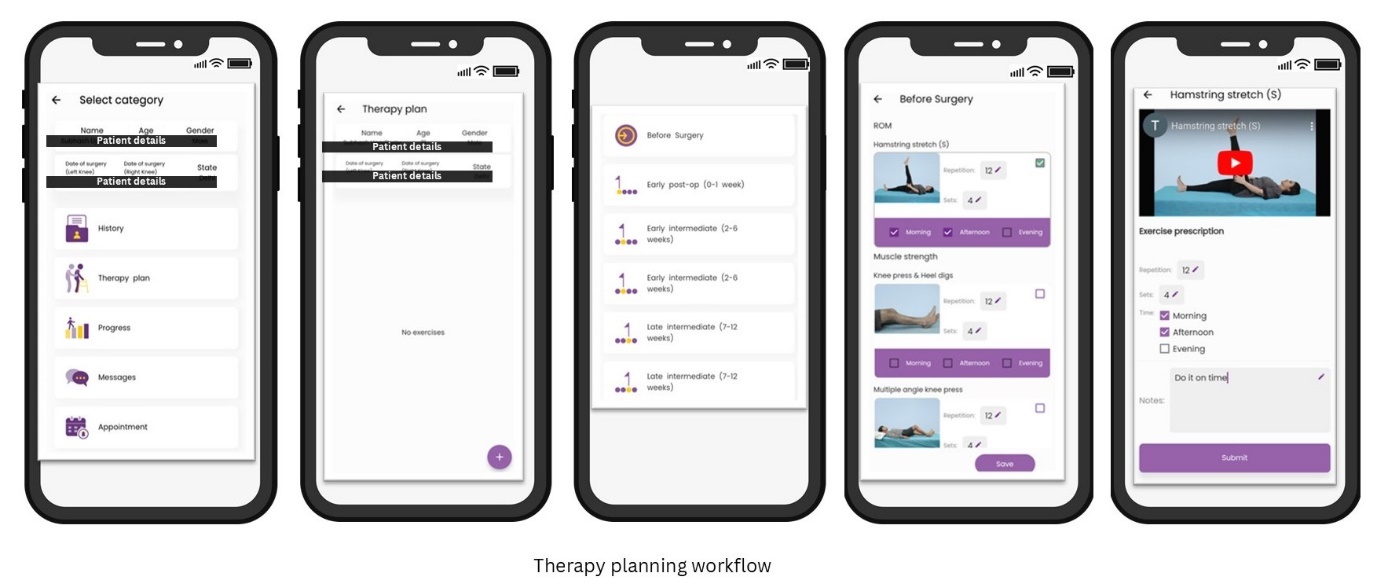


**
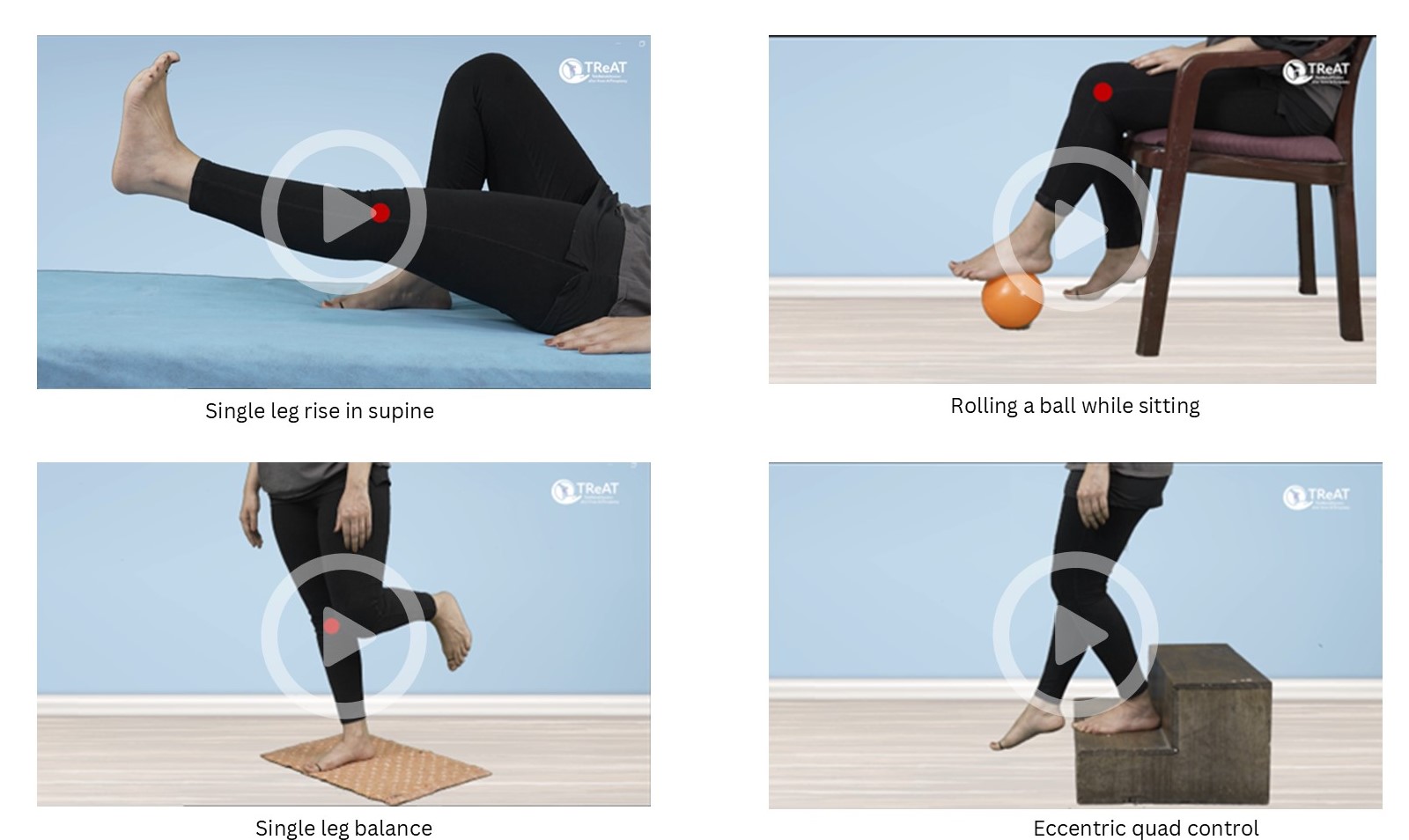
**

A repository of 46 exercise videos targeted at strengthening lower limb muscles improving range of motion, endurance and function are available for physiotherapists across different stages of rehabilitation. The physiotherapist can assign these exercise videos along with notes on the number of repetitions, number of times exercise is to be performed which can be viewed in the patient interface.

eFigure 4 My diary functionality
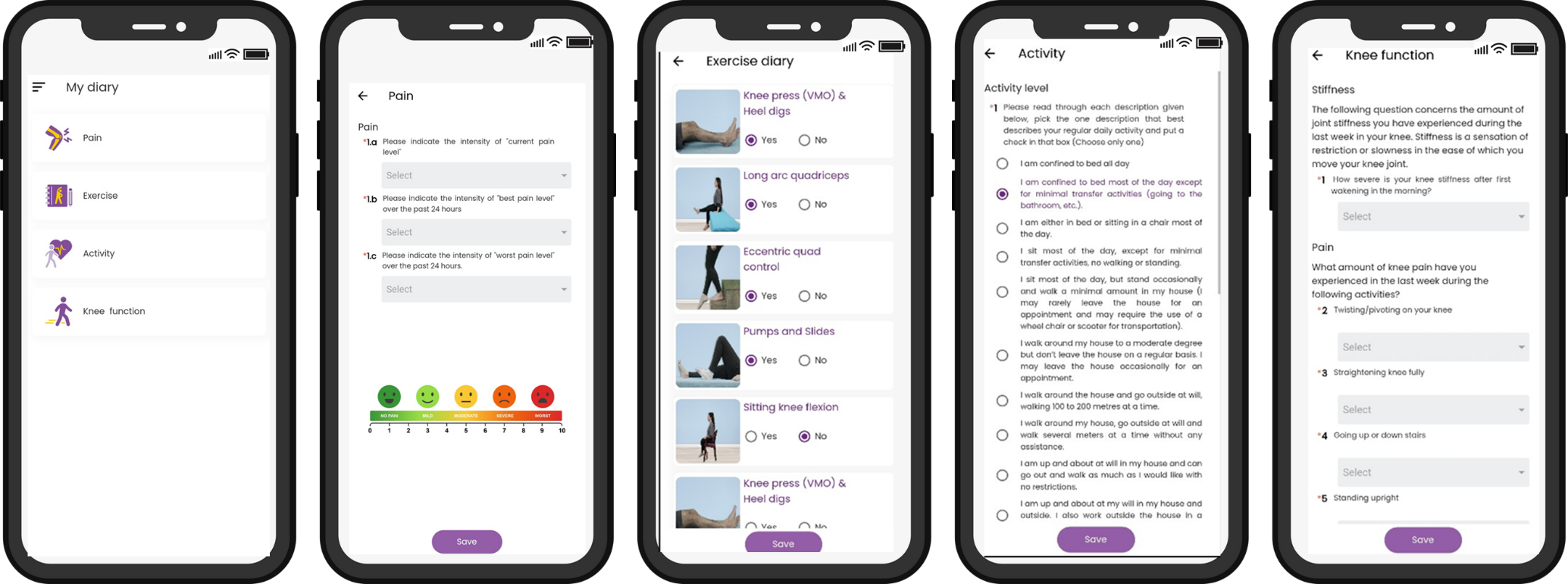


Participants are expected to complete activity and knee function diary every fortnight

Participants are expected to complete pain and exercise diary daily

eFigure 5 Progress graphs for monitoring


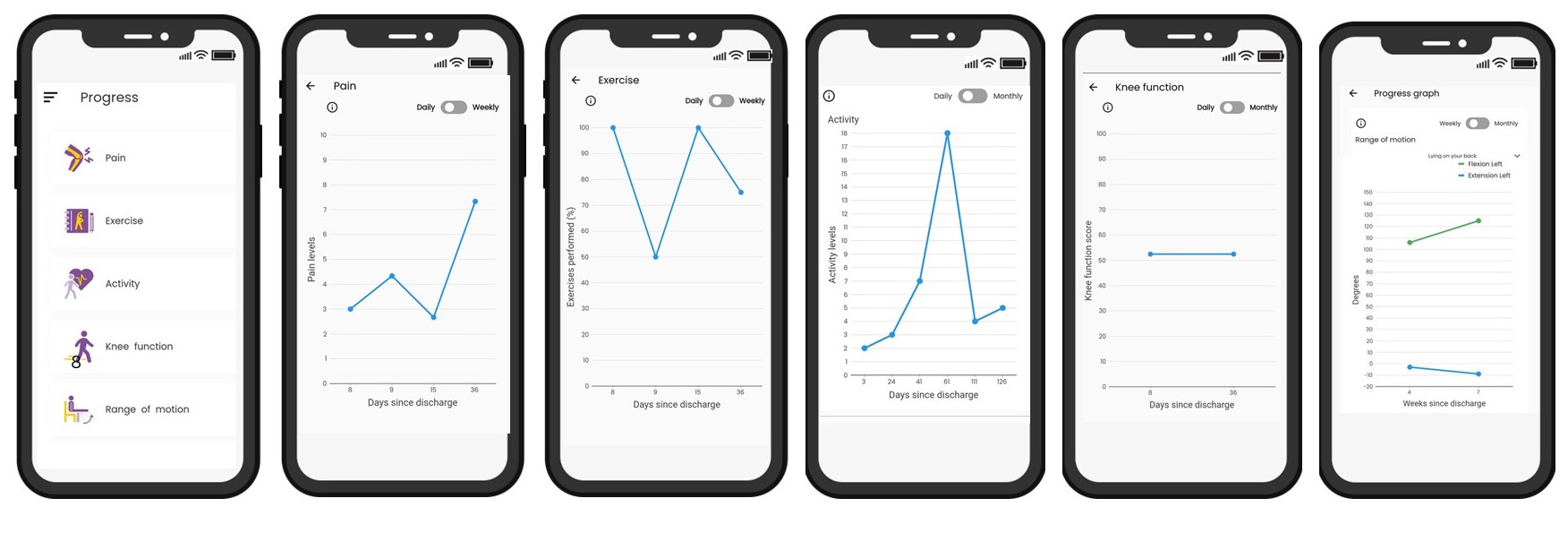


Healthcare providers, patients and their family members can view the progress graphs, with an option to modify the horizontal time scale to either days or week since discharge.

eTable 1 Motivational messages (Participant)

Number of messages (excluding conditional messages) sent to patients and family members are 38

| **Theme** | **To whom** | **Timing** | **Content (English)** | **Content (Hindi)** |
| --- | --- | --- | --- | --- |
| Welcoming to the app | Patient | After first time login | Welcome to the Knee Rehabilitation program | घुटने के पुनर्वास कार्यक्रम में आपका स्वागत है। |
|  | Family member | After first time login | Welcome to the Knee Rehabilitation program! You will be able to see the progress of (patient name), and doctor appointments. | घुटने के पुनर्वास कार्यक्रम में आपका स्वागत है! आप (रोगी का नाम) की प्रगति, और डॉक्टर के साथ उनके अपॉइंटमेंट देख सकेंगे। |
| Pain | Patient | Once in every 48 hours for 2 weeks after date of surgery | Namaste (Patient Name) Knee pain is normal in the first few weeks after surgery. We hope you are taking the pain medications, applying ice, and exercising regularly. | नमस्ते (रोगी का नाम) सर्जरी के बाद पहले कुछ हफ्ते घुटने में दर्द होना सामान्य है। हमें उम्मीद है कि आप दर्द की दवाई ले रहे हैं, बर्फ से सिकाई कर रहे हैं और नियमित रूप से व्यायाम कर रहे हैं। |
| Exercise | Patient | Start a day after date of discharge Frequency: weekly once for 3 months | Namaste (Patient Name) Don’t forget to do your EXERCISES. They are necessary for your recovery. | नमस्ते (रोगी का नाम) व्यायाम करना न भूलें। वे आपके ठीक होने के लिए आवश्यक हैं। |
| Being connected | Patient | Start 2 weeks after date of discharge Frequency: every month for 3 months | Namaste (Patient Name) We hope your knee pain is better and you can do the exercises. If you have any concerns, please send a message to your doctor. | नमस्ते (रोगी का नाम) हमें उम्मीद है कि आपके घुटने का दर्द कम है और आप व्यायाम कर पा रहे हैं । यदि आपको कोई भी परेशानी है तो कृपया अपने डॉक्टर को मैसेज करें l |
| Danger sign | Patient | Send on the 10th day from the date of surgery | Namaste (Patient Name) Keep the wound dry! If you notice discharge (pus and bloody) or too much pain or excessive redness, please take a photo and send to your doctor. | नमस्ते (रोगी का नाम) घाव को सूखा रखें! यदि घाव से मवाद या खून आ रहा है या बहुत अधिक दर्द हो रहा है या घाव के आसपास अत्यधिक लालिमा दिख रही है, तो कृपया फोटो लें और डॉक्टर को भेजें। |
| Move | Patient | Send on 12th day from the date of surgery. | Namaste (Patient Name) Walk as much as you can within your home! | नमस्ते (रोगी का नाम) जितना हो सके अपने घर के अंदर चलें ! |
| Move | Patient | First message should start after 15 days from the date of discharge. Send every fortnightly until 3 months. | Namaste (Patient Name) Do you know climbing stairs is a way of exercise? Use the railing while using the staircase! | नमस्ते (रोगी का नाम) क्या आप जानते हैं सीढ़ियां चढ़ना व्यायाम का एक तरीका है? सीढ़ी का उपयोग करते समय रेलिंग को पकड़ें! |
| Move | Patient | Start after 45th day from the date of surgery. Send every fortnight till 3 months. | Namaste (Patient Name) Try to STEP OUT of your home and go for a walk during daytime! | नमस्ते (रोगी का नाम) दिन में अपने घर से बाहर निकलें और चलें। |
| Scheduled appointment | Patient | When the Physiotherapist/surgeon fixes an appointment with the patient | Namaste (Patient name) Your consultation via video call is fixed by (Physiotherapist/Surgeon name) on dd/mm/yyyy at time HH: MM (12 hr format) (AM/PM)) | नमस्ते (रोगी का नाम) आपका अपॉइंटमेंट (फिजियोथेरेपिस्ट/सर्जन नाम) के साथ वीडियो कॉल के माध्यम से dd/mm/yyyy को HH: MM (12 घंटे का प्रारूप )(AM/PM)) बजे तय किया गया है। |
|  | Family member | When the Physiotherapist/surgeon fixes an appointment with the patient | Namaste,(Patient name) consultation via video call with (Physiotherapist/surgeon name) is fixed on dd/mm/yyyy at time HH: MM (12 hr format (AM/PM)) | नमस्ते (रोगी का नाम) का अपॉइंटमेंट (फिजियोथेरेपिस्ट/सर्जन नाम) के साथ वीडियो कॉल के माध्यम से dd/mm/yyyy को HH: MM (12 घंटे का प्रारूप (AM/PM)) बजे तय किया गया है। |
| Appointment reminder | Patient | Reminder notification before 30 minutes of scheduled appointment | Namaste (Patient name), Your video consultation with Dr (name) is due at HH: MM (12 hr format (AM/PM)). Be ready for the call. | नमस्ते (रोगी का नाम), आपका वीडियो कॉल HH: MM (12 घंटे का प्रारूप (AM/PM)) बजे डॉ (फिजियोथेरेपिस्ट/सर्जननाम) के साथ तय है। कॉल के लिए तैयार रहें। |
|  | Family member | Reminder notification before 30 minutes of scheduled appointment | Namaste, (Patient name) video consultation with Dr (name) is due at HH: MM (12 hr format (AM/PM)). Be ready for the call. | नमस्ते, (रोगी का नाम) का वीडियो कॉल HH: MM (12 घंटे का प्रारूप (AM/PM)) बजे डॉ (फिजियोथेरेपिस्ट/सर्जननाम) के साथ तय है।कॉल के लिए तैयार रहें। |
| Therapy plan | Patient | When the therapy plan is updated by Physiotherapist/surgeon | Namaste (Patient name), Your exercises have been updated. Please check and follow as prescribed. | नमस्ते (रोगी का नाम), आपके व्यायाम अपडेट किए गए हैं । कृपया बताए अनुसार पालन करें। |
| Reminder for pain e-diary | Patient | Next day from the date of discharge | We want to know how you are doing! Can you rate your knee pain in My Diary? | हम जानना चाहते हैं कि आप कैसे हैं? क्या आप मेरी डायरी में अपने दर्द के स्तर का मूल्यांकन कर सकते हैं? |
| Reminder for pain e-diary- I | Patient | For first 6 weeks after discharge, if no pain score reported on consecutive 3 days. | We hope you are getting better. It’s been 3 days since we heard from you. Can you rate your knee pain in My Diary? | हमें आशा है कि आप बेहतर हो रहे हैं। हमें आपसे सुने हुए तीन दिन हो गए हैं। क्या आप मेरी डायरी में अपने घुटने के दर्द का मूल्यांकन कर सकते हैं? |
|  | Family member | For first 6 weeks after discharge, If no pain score reported on consecutive 3 days. | It’s been 3 days since (Patient name), entered their pain score. Please encourage (Patient name) to rate their knee pain so that we know how they are recovering. | (रोगी का नाम) ने तीन दिन से अपने दर्द के स्तर को नहीं भरा है। कृपया (रोगी का नाम) को दर्द के स्तर को रेट (मूल्यांकन) करने के लिए प्रोत्साहित करें ताकि हम जान सकें कि वे कैसे हैं। |
| Reminder for pain e-diary- II | Patient | After 6 weeks from DOD- If no pain score reported in the last 2 weeks. | We hope you are getting better. It’s been a while since you completed your pain e-diary. | हमें उम्मीद है कि आप बेहतर हो रहे हैं। आपको अपनी दर्द ई-डायरी को पूरा किए हुए काफी समय हो गया है। |
|  | Family member | After 6 weeks of DOD- If no pain score reported in the last 2 weeks | It’s been a while since (Patient name) has entered their pain score. Please encourage (Patient name) to rate the pain levels so that we know how they are doing. | (रोगी का नाम) ने काफी समय से अपने दर्द के स्तर को मेरी डायरी में नहीं भरा है। कृपया (रोगी का नाम) को दर्द के स्तर को रेट करने के लिए प्रोत्साहित करें ताकि हम जान सकें कि वे कैसे हैं। |
| Reminder for activity and knee function e-diary | Patients | On 15th day after the date of surgery. | We hope you have started walking inside your home.  To help us monitor your progress, please complete your activity and knee function regularly in the e-dairy. | हमें उम्मीद है कि आपने घर के अंदर चलना शुरू कर दिया है। आपकी प्रगति की निगरानी करने के लिए, कृपया ई-डेयरी में नियमित रूप से अपनी गतिविधि और घुटने के कार्य को पूरा करें। |
|  | Family member | On 15th day after the date of surgery | We are remotely monitoring how (Patient name) is doing. It will be helpful if you encourage (Patient name) to complete their activity and knee function e-dairy regularly. | हम दूर से निगरानी कर रहे हैं कि (रोगी का नाम) की रिकवरी कैसी है । कृपया आप (रोगी का नाम) को उनकी गतिविधि और घुटने के कार्य को नियमित रूप से ई- डायरी में भरने के लिए प्रोत्साहित करते रहें। |
| Reminder for Exercise e-diary | Patients | Start date- When physiotherapist starts assigning exercises. Send if exercise diary is not filled in consecutive 3 days till 3 months | It’s been 3 days since you completed your exercise e-diary. We hope you are doing your exercises regularly. | आपको अपना व्यायाम-डायरी भरे हुए 3 दिन हो चुके हैं। हम आशा करते हैं कि आप अपने व्यायाम नियमित रूप से कर रहे हैं। |
|  | Family member | Start date- When physiotherapist starts assigning exercises. Send if exercise diary is not filled in last 3 days till 3 months | It’s been 3 days since (Patient name) has completed their exercise diary. Please check with them if they are doing their exercises regularly. | (रोगी का नाम) ने 3 दिन से व्यायाम-डायरी नहीं भरा है। कृपया देखें कि क्या वे नियमित रूप से व्यायाम कर रहे हैं? |
| Activity progress rewards | Patients | If patient scores more than the previous activity level. For e.g.: Previous level was 5, but in the next time patient scored 6. | Well done! You seem to be more active. Keep it up! | बहुत बढ़िया! ऐसा लगता है कि आप सक्रिय हो गए हैं। इसे जारी रखें! |
| Exercise progress rewards in a day | Patient | When patient scores more than 75% in a day | Well done (Patient name)! Today you have completed most of the exercises. Keep it up! | बहुत बढिया (रोगी का नाम)! आज आपने अधिकांश व्यायाम पूरे किए हैं। इसे जारी रखें! |
|  | Family member | When patient scores more than 75% in a day | Today (Patient name) has completed most of the exercises. Keep encouraging them to continue exercising. | आज (रोगी का नाम) ने अधिकांश व्यायाम पूरे किए हैं। उन्हें व्यायाम करते रहने के लिए प्रोत्साहित करते रहें। |
| Exercise progress rewards in a week | Patient | When patient performs less than 75% exercises in a week | Well done (Patient name) for doing your exercises. You will benefit more if you do it daily and follow all the exercises as prescribed. | व्यायाम करने के लिए आपको बधाई! अगर आप व्यायाम हरदिन बताए अनुसार करेंगे तो आपको अधिक लाभ होगा। |
|  | Patient | When patient performs no exercises in a week. | It’s been a week since you completed your exercise diary. We hope you are doing your exercises. | आपको अपना व्यायाम-डायरी भरे हुए एक सप्ताह हो गया है। हमें उम्मीद है कि आप व्यायाम कर रहे हैं। |
| Last day | Patients | When the healthcare provider decides to complete the treatment regimen. | Congratulations! You have completed the rehabilitation program. We hope it was helpful. Please continue to follow the advice of your doctor. Thank you for your participation. | बधाई हो! आपने पुनर्वास कार्यक्रम पूरा कर लिया है. हमें उम्मीद है कि यह मददगार रहा होगा। कृपया अपने डॉक्टर की सलाह का पालन करना जारी रखें। आपकी भागीदारी के लिए धन्यवाद। |

eFigure 6 Messaging and video consultation functionality


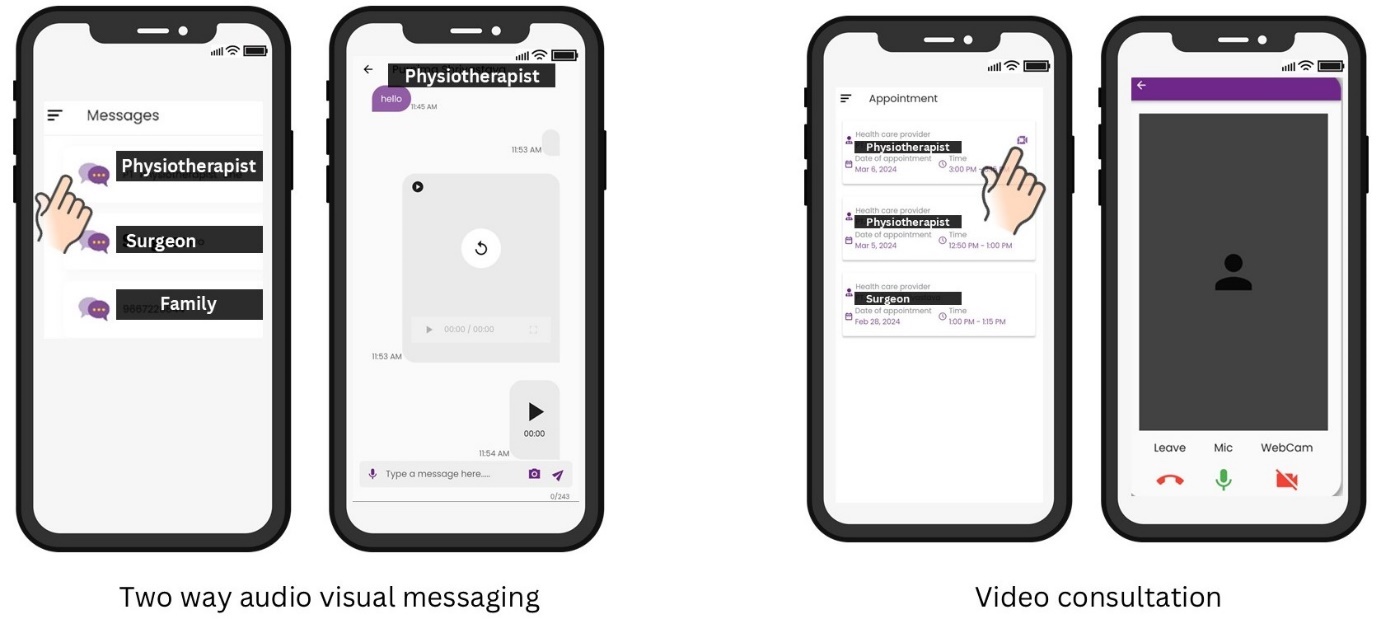


Patients and family member can message the physiotherapist or surgeon through the TReAT app, and communicate their concerns through text, audio, or video messages. They can request for a video consultation, which the healthcare provider can arrange as per their availability. Participants can join the scheduled video consultation either through the patient interface or the family member interface.

eFigure 7 Therapy planning posters


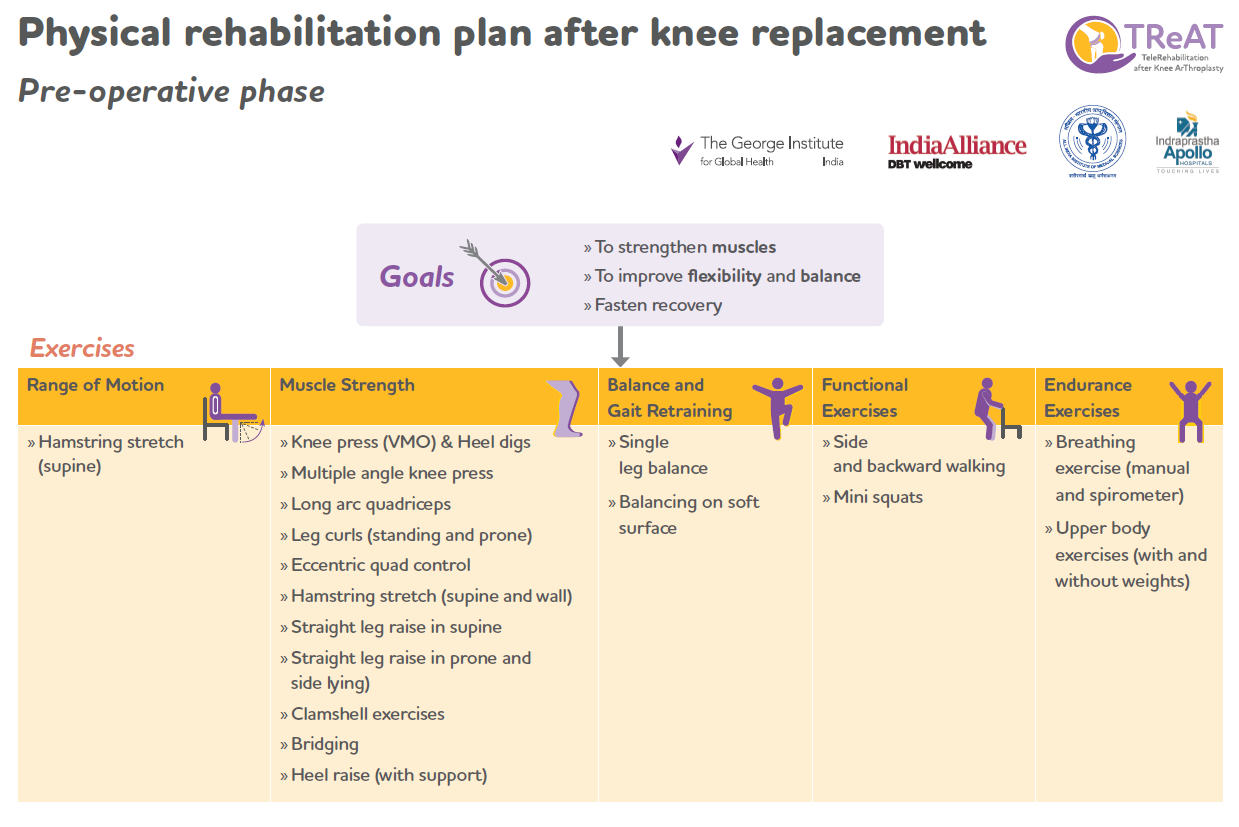


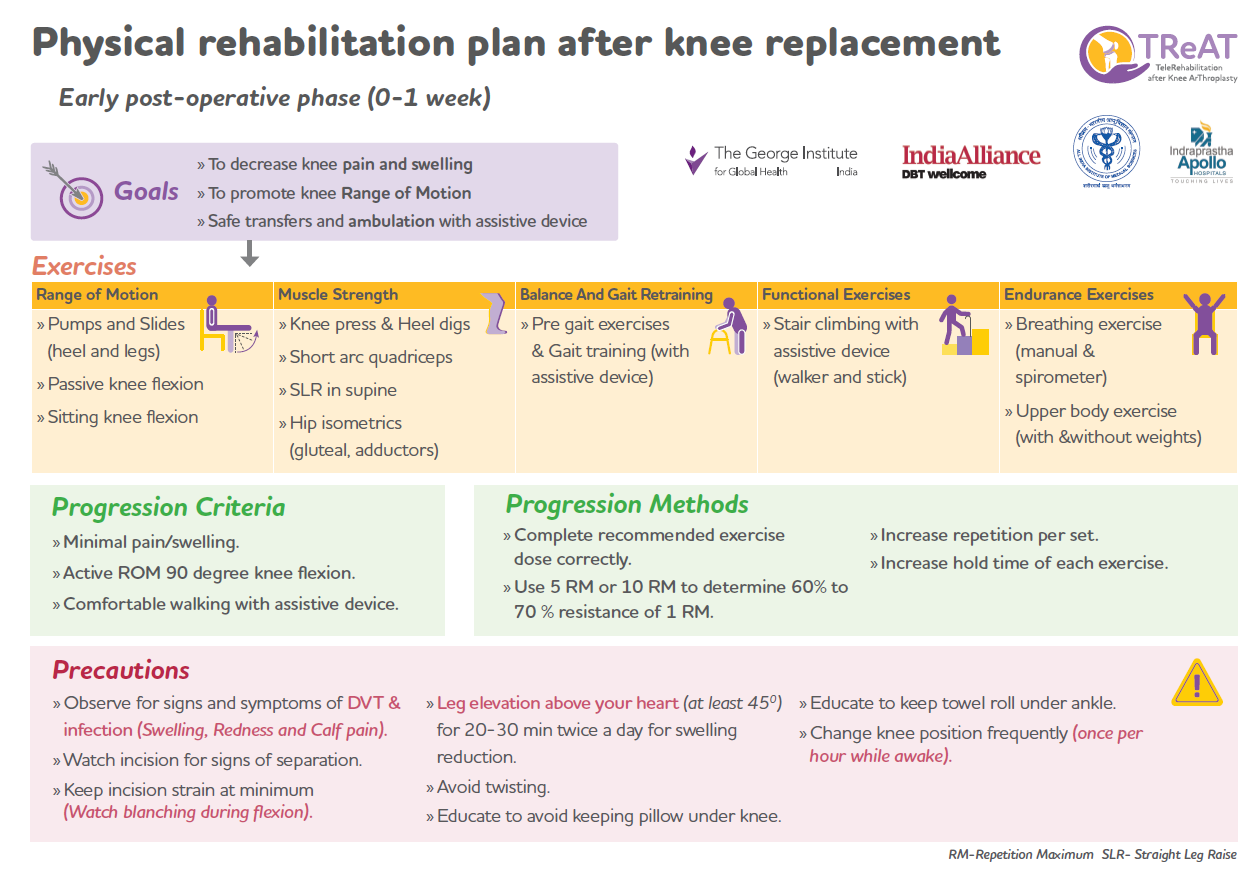

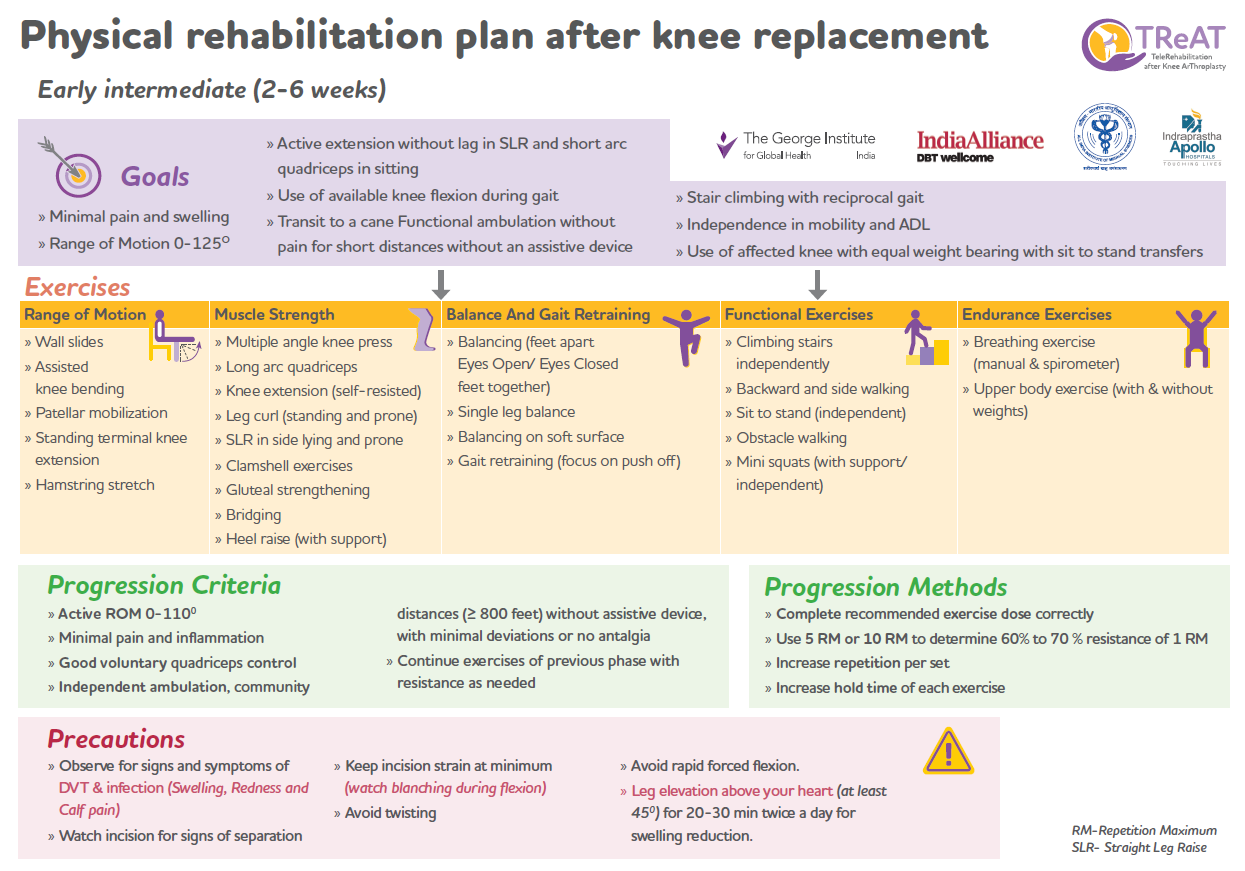

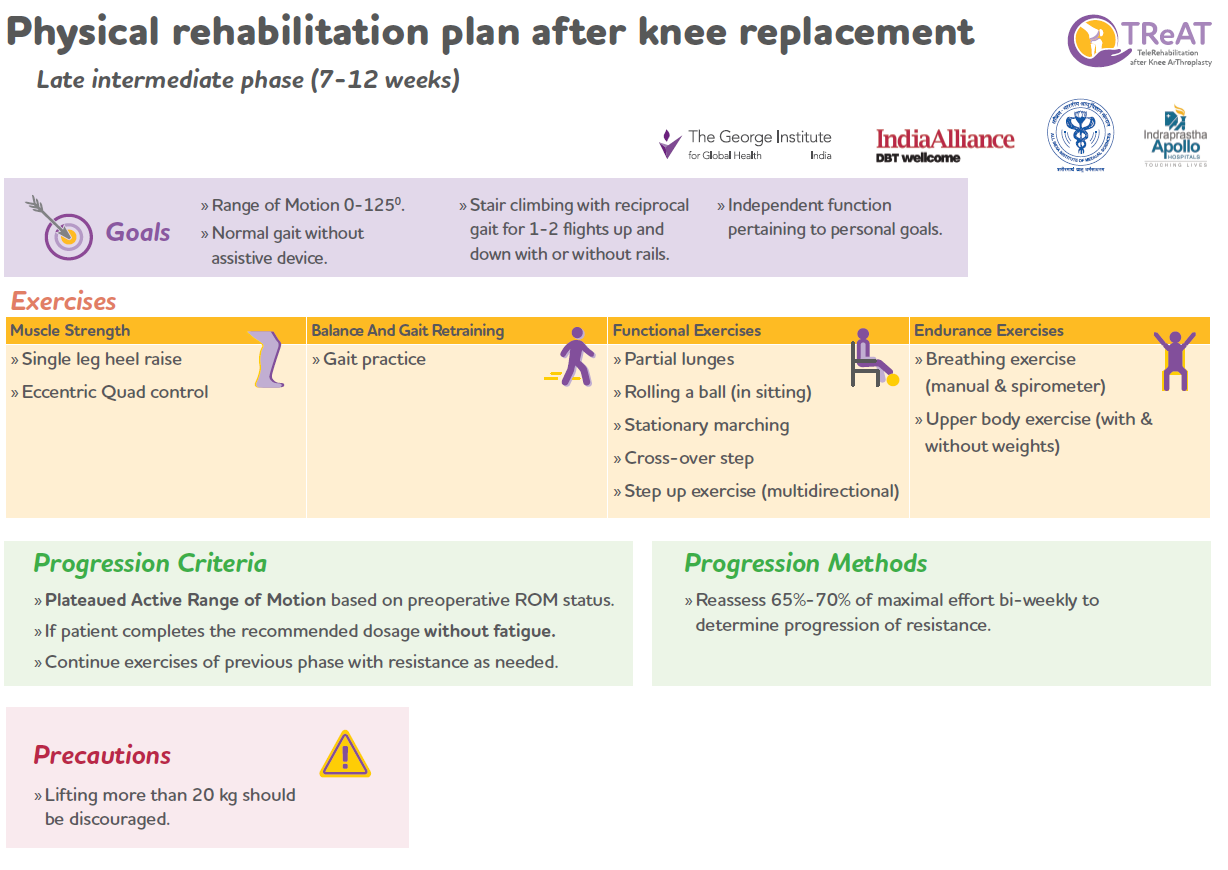


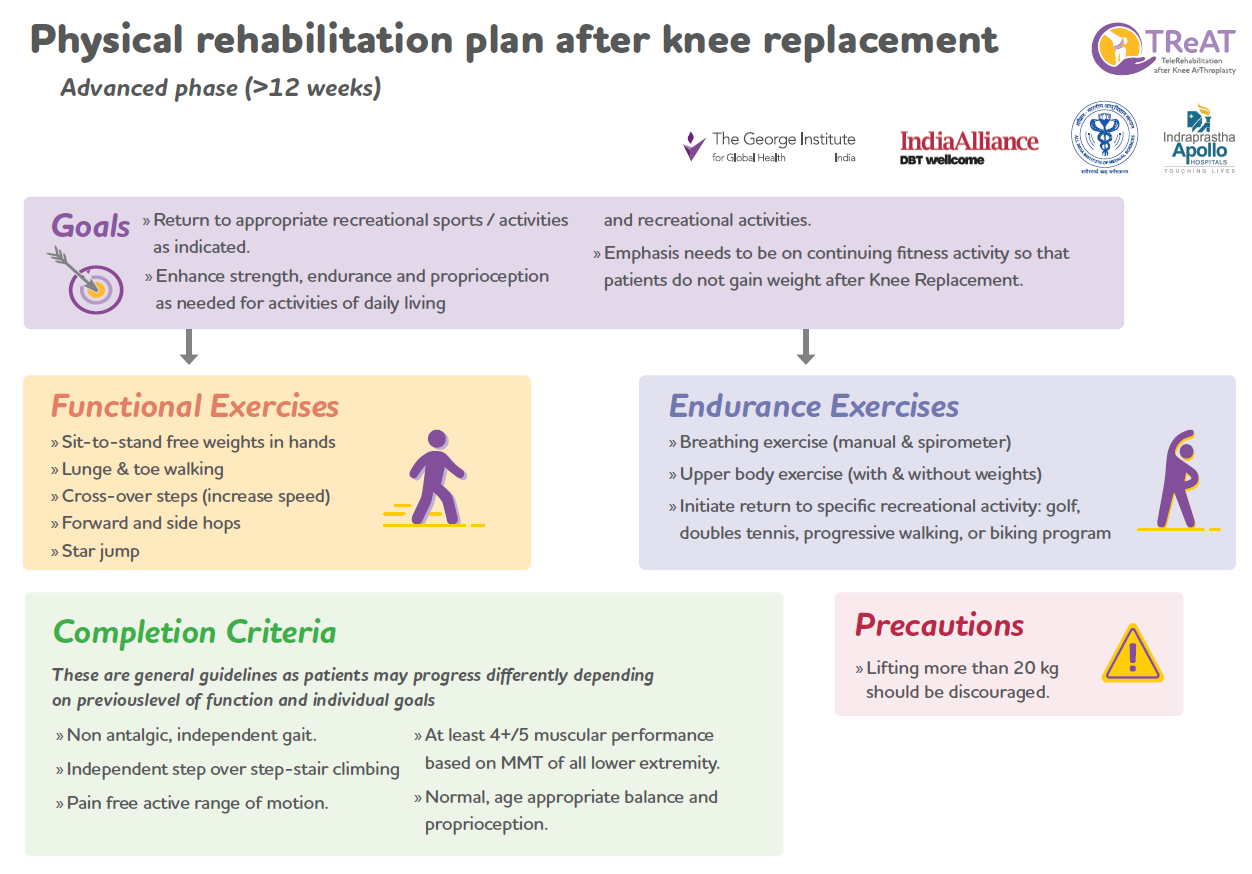

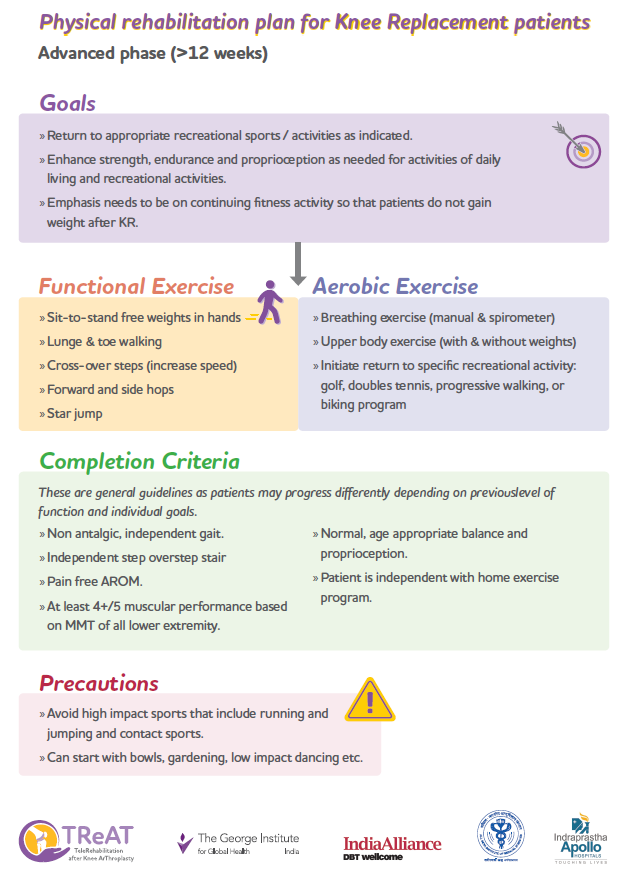


eFigure 8 Standardised instructions for conducting performance-based functional tests


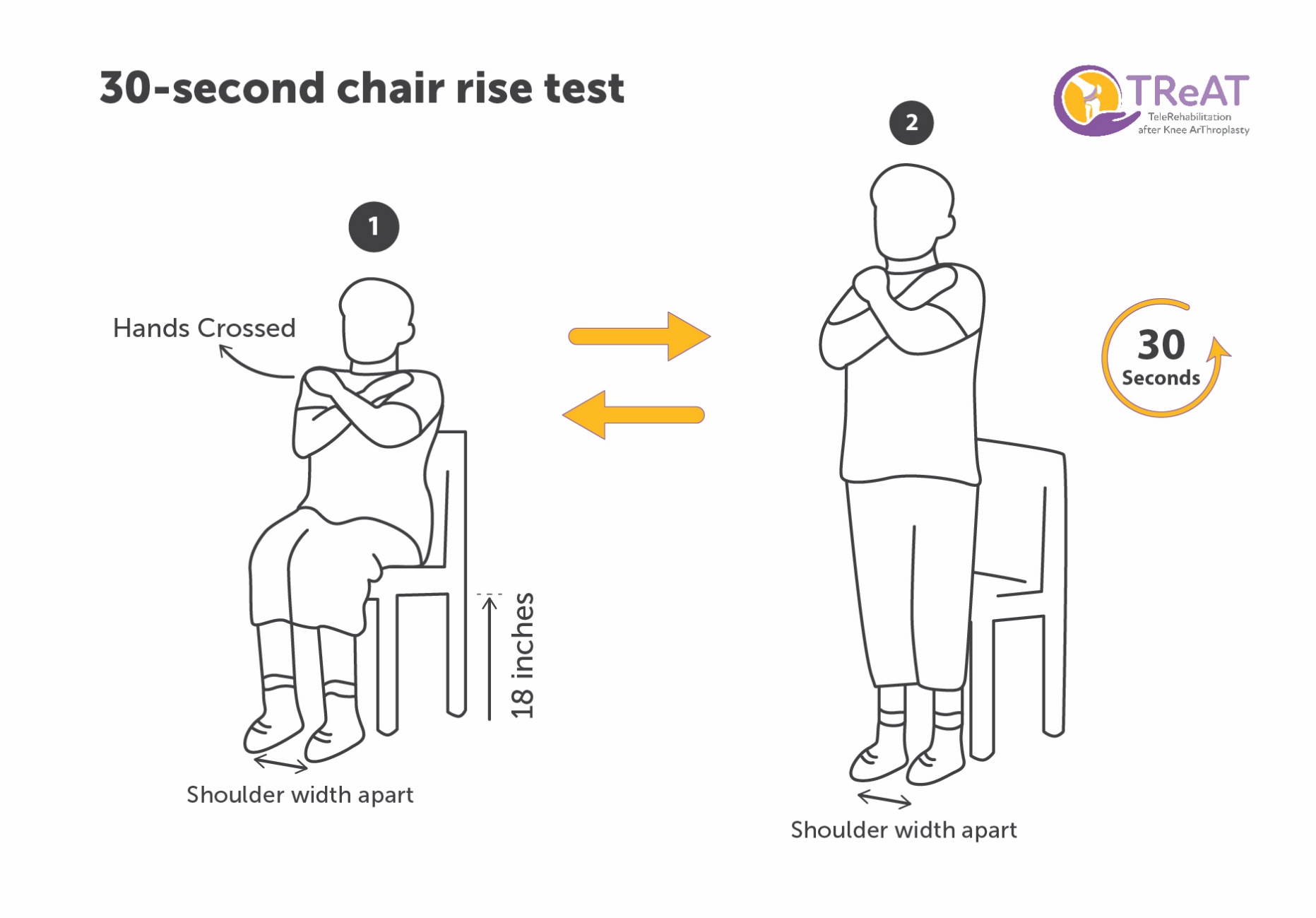


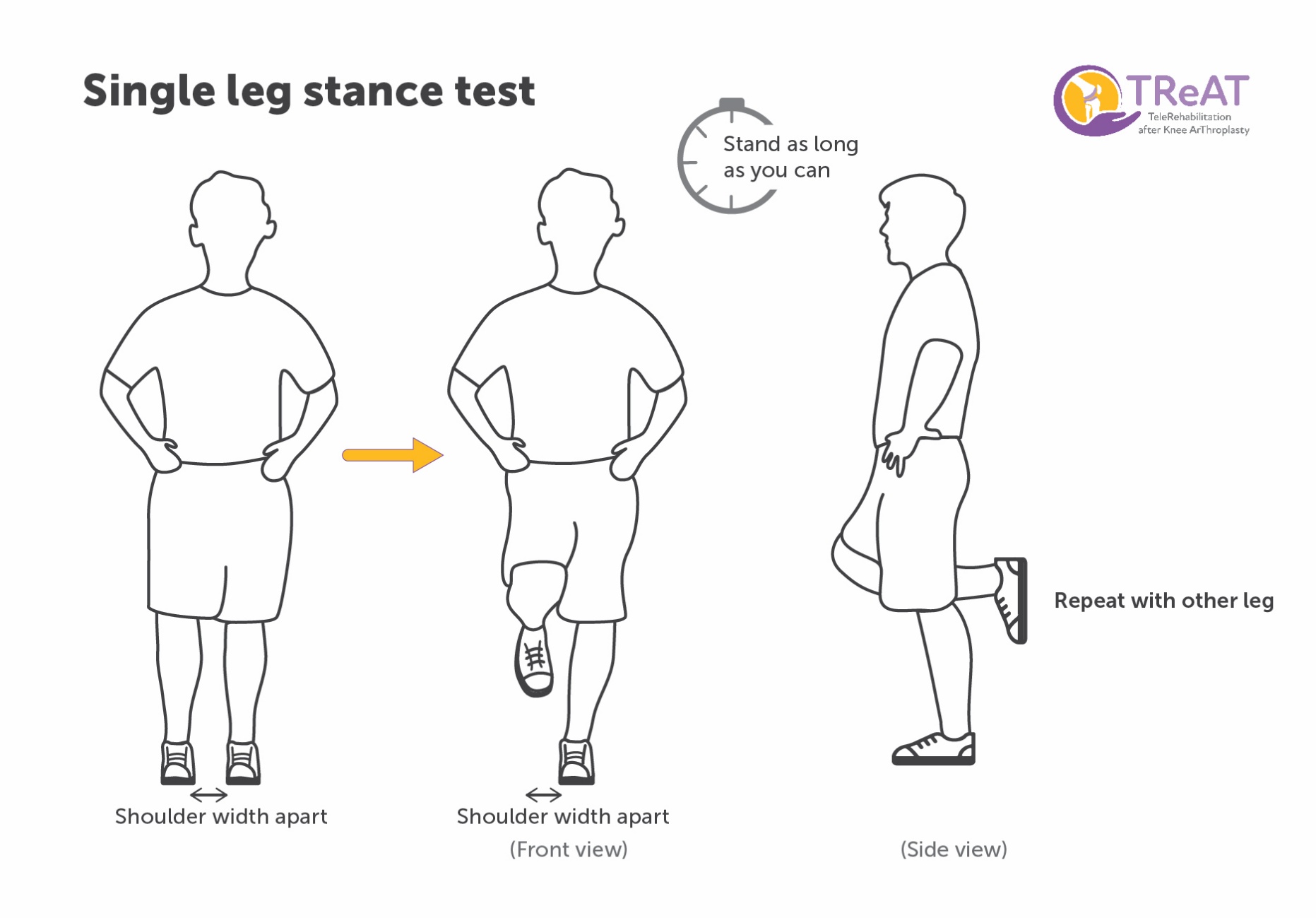


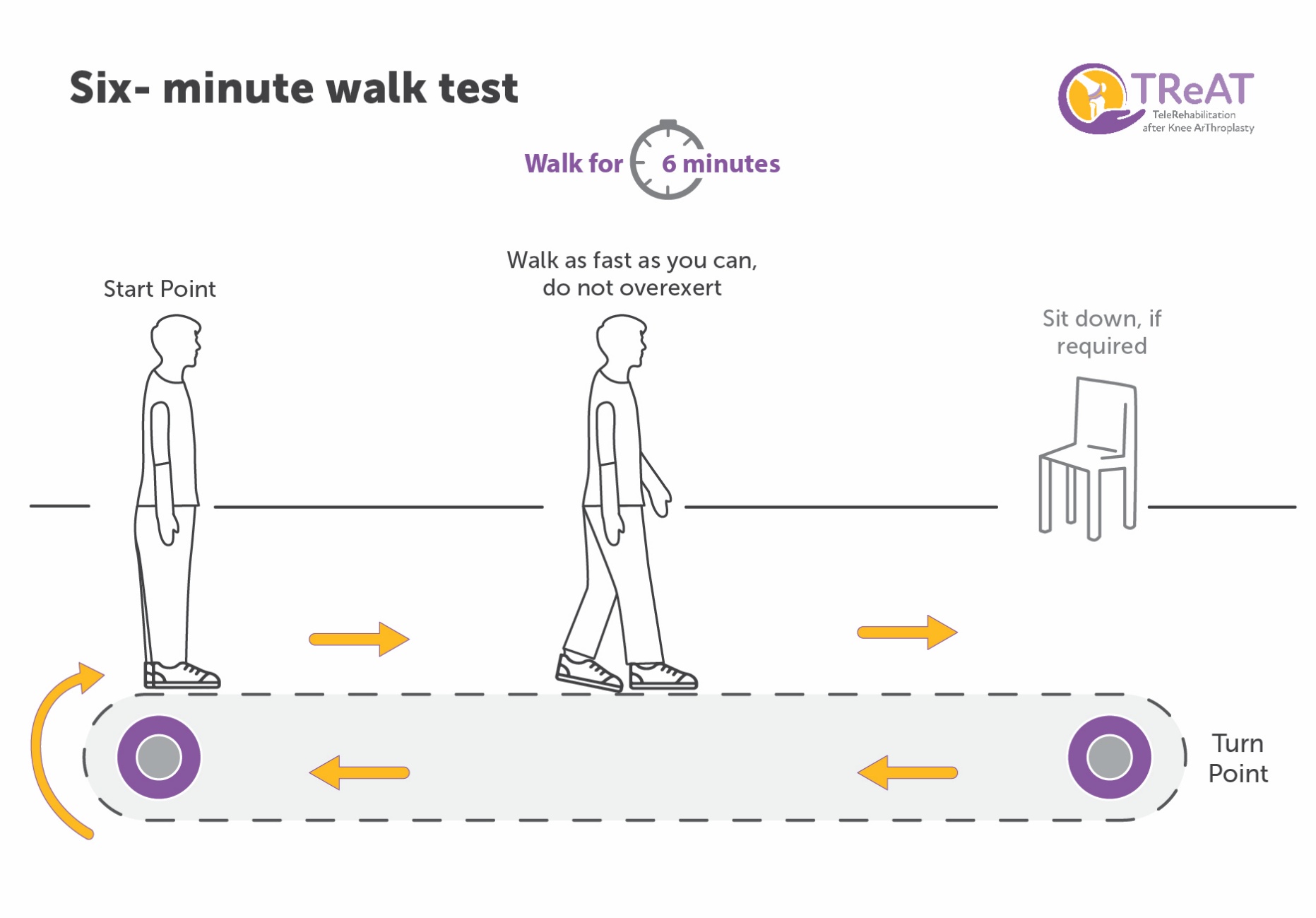


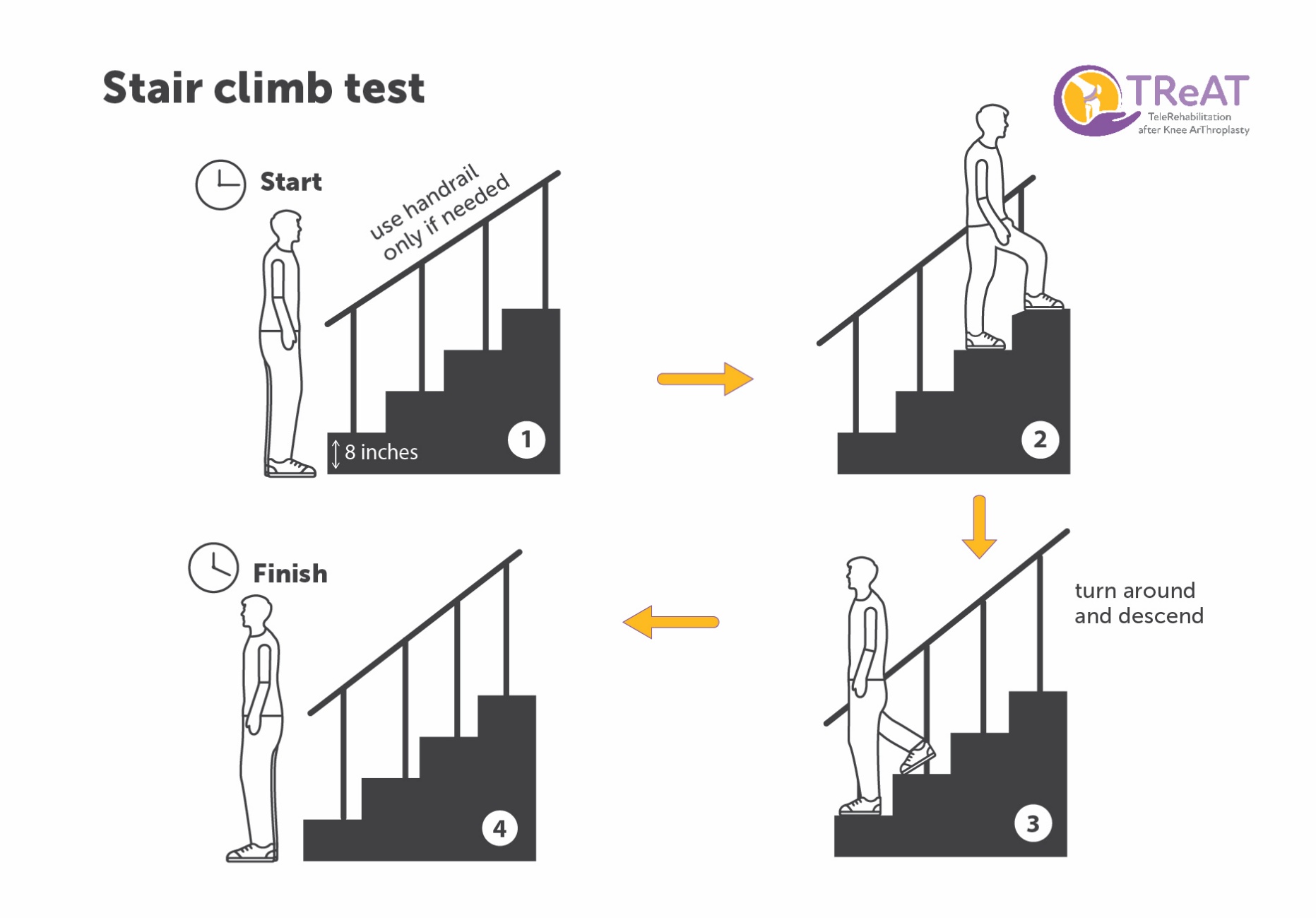


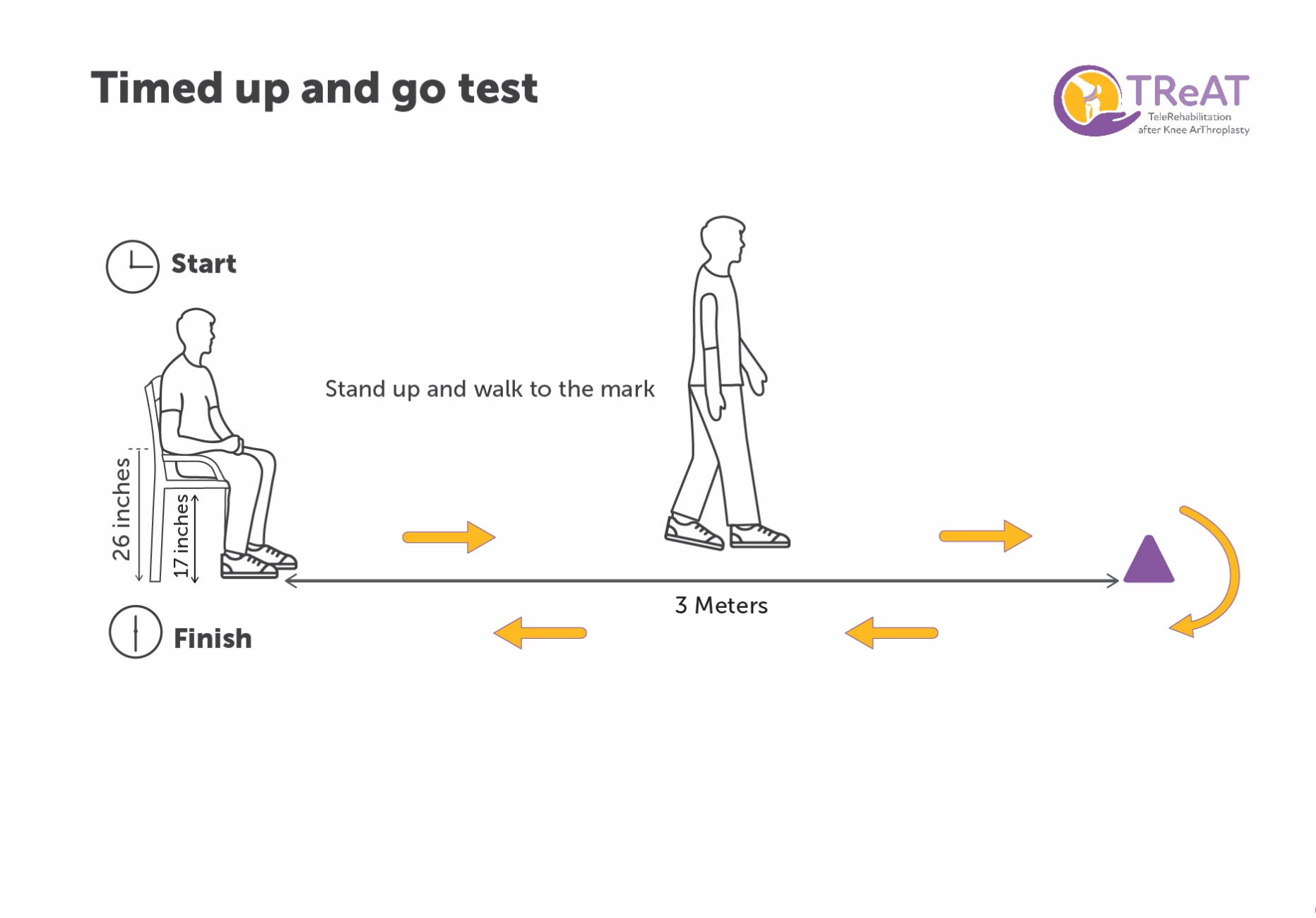

Supplement: online supplemental file 1 [file bmjopen-15-11-s001.docx]
