# Supplementary material for: How effective is mHealth-supported home-based post-knee arthroplasty rehabilitation in improving knee function and continuum of care: protocol of an open label randomised controlled trial from India
Source: BMJ Open. 2025 Nov 26;15(11):e106469. doi: 10.1136/bmjopen-2025-106469 (PMC12658543; doi:10.1136/bmjopen-2025-106469)
Supplement: Supplementary file 2 [file bmjopen-2025-106469.reviewer_comments.pdf]

PEER REVIEW HISTORY

BMJ Open publishes all reviews undertaken for accepted manuscripts. Reviewers are asked to complete a checklist review form and are provided with free text boxes to elaborate on their assessment. These free text comments are reproduced below.

ARTICLE DETAILS

Title (Provisional)

How effective is mHealth supported home-based post knee arthroplasty rehabilitation in improving knee function and continuum of care: protocol of an open label randomized controlled trial from India

Authors

Girotra, Siaa; Shrivastava, Purnima; Kumar, Ajit; Madan, Ruchika; Grover, Seema; Batra, Sahil; Garg, B; Praveen, Devarsetty; Chatterjee, Susmita; Kasotia, Ankita; Biswas, Shyamashree; Soni, Manoj; Gara, Sridevi; Malhotra, Rajesh; Maddison, R; Devasenapathy, Niveditha

---

VERSION 1 - REVIEW

**Reviewer**	**1**
**Name**	**Hailey, David**
**Affiliation**	**University of Wollongong, -**
**Date**	**26-Jul-2025**
**COI**	**None**

Appropriate points are made on the challenges for management of the patient population requiring knee arthroplasty. And also of the possible limited generalizability of the study findings.

The approach taken gives a basis for decisions in health care and for future investigations of these treatments.

**Reviewer**	**2**
**Name**	**Nae, Jenny**
**Affiliation**	**Lund University, Department of Health Sciences**
**Date**	**03-Sep-2025**
**COI**	**None**

The aim with this upcoming study was to compare app-supported home-based rehabilitation versus home-based rehabilitation in patients undergoing knee arthroplasty. I found this study protocol very interesting, and the clinical value of this intervention is high as it targets the need for rehabilitation support in unsupervised home-based physical rehabilitation as well as a technical solution for monitoring progress.

Overall comment:

My biggest concern is that the intervention includes so many different features in the app which might be overwhelming for the study population to be included in this study. Has the app been developed and together with patients and caregivers? If so, was the usability of the app evaluated by the patients? I miss more information and discussion about this.

Another overall comment is whether this study investigate implementation in real world scenarios or just in a research context, see specific comment under methods.

ABSTRACT:

1. To get a better understanding of the methods I would like a small description of what usual care means in this study.

INTRODUCTION:

1. Page 4, lines 20-21: It would help the reader if you included a definition of what internet-based telerehabilitation is.

2. Page 4, lines 56-57: Could you describe this study more, the results as well as elaborate a bit more about what changes you did after the pilot testing.

3. Page 4, lines 30-40: There are other programs around the world with different digital management programs for hip and knee osteoarthritis (OA) for example. I am missing more results and rationale around why digital/telerehabilitation can be a preferable solution for some patients. See for example a study by Cronström et al: "I would never have done it if it hadn't been digital': a qualitative study on patients' experiences of a digital management programme for hip and knee osteoarthritis in Sweden"

4. I am missing some background around the frameworks you are using for complex interventions, the framework by Linnan and Stecker. I think it would help understand the reasoning of your study to include a small section on this in the introduction and not only in the objectives.

5. Objectives: Be consistent in what you call the control condition. In the Primary objectives you call it "usual home-based rehabilitation" and in the secondary objective "usual care"

METHODS:

6. Trail design: The intervention is planned to be open-labeled. I understand that it is difficult to blind the interventions but are there any things you could do to increase the possibility of

blinding the participants, for example be vaguer in the information letter to participants so that they do not know what the two options are? I would like some discussion about this.

7. Trail design: again about the consistency in what you call the control condition, here you call it “usual care home-based rehabilitation”, see also comment under objectives. My suggestion is that you choose one way of reporting this and use it throughout the manuscript.

8. Exclusion criteria 2: “Individuals unable to make a hospital visit at 3 and 6 months after surgery”. Could you clarify the rationale behind this exclusion criterion? I’m concerned that excluding individuals who, for example, live far away or face economic barriers to participation may reduce the generalizability of the study findings. Couldn’t they be included for some parts of the study, for example PROMs?

9. Page 7, line 15: “Control intervention”. See comments under objectives and methods about what to call the control intervention, be consistent with terminology to make it easier for the reader.

10. Page 7, line 32: “Test intervention”, I suggest adding app-based rehabilitation since that is what you refer to in the objectives.

11. Could you clarify how the physical therapists are recruited. This is unclear to me, and how many physical therapists there are for the app-based rehabilitation to give support via the app and create the therapy planning.

12. Page 10, lines 50-60: A research coordinator will be available for technical help with the app both on-site in the beginning as well as doing check-in phone calls to assist with the app. My concern regarding this approach is that you evaluate whether the research project works with all this support and then you do not know if the intervention works later when the support is not there anymore when the project stops. Have you thought about this and maybe not providing this service, hence only see how the intervention works without this support? We know from, for example, prevention studies that injuries decrease when the physical therapists are in teams helping during studies with the prevention training but when the research stops the injuries increase again. So, the most important question is how do we implement this so that it works in the real world?

13. Page 11: “Blinding”, please clarify if the research coordinator performing the data collection of the outcomes will be blinded to the interventions, this should be possible and are highly recommended.

14. Page 11: “Outcomes”, Why are you planning to distribute the KOOS questionnaire on paper and not through for example REDCap? By using REDCap you could include patients who are not able to come to the follow-up visits but still be able to answer questionnaires online.

15. Page 12: Secondary outcomes, there is a need for a more detailed description of the physical tests, as it is now it is not possible to reproduce the study method without going into each of the reported references, which is inconvenient for the reader.

16. Page 12: Please write out the explanation of the different secondary outcomes in the text and not only under Table 2. It makes it difficult to read when it is under the table. And, please provide them with appropriate references for the outcomes when those are missing.

17. Page 13, "Process outcomes" lines 23-27. It is difficult to understand how you are going to measure self-efficacy to rehabilitation, this need more description.

18. Page 13: Economic component. Please specify if that this is in both groups.

19. Page 14, lines 2-13: Why are you using this approach and not collecting the data online directly via REDCap?

20. Page 14, lines 57-60: Again, if using the online REDCap this problem is solved.

21. Page 15, Public involvement: I think this is important information that you have had patints testing the app before this trail and I missed this information earlier in the manuscript. I think it would be nice if you could add more description of this and results from this study on page 7 "Test intervention" to clarify this earlier for the reader.

VERSION 1 - AUTHOR RESPONSE

Reviewer 1

Dr. David Hailey, University of Wollongong
Comments to the Author:
Appropriate points are made on the challenges for management of the patient population requiring knee arthroplasty. And also of the possible limited generalizability of the study findings.
The approach taken gives a basis for decisions in health care and for future investigations of these treatments.
Response: *We thank the reviewer for taking time to review the manuscript and provide their helpful feedback.*

Reviewer 2

Dr. Jenny Nae, Lund University
Comments to the Author:
1. The aim with this upcoming study was to compare app-supported home-based rehabilitation versus home-based rehabilitation in patients undergoing knee arthroplasty. I found this study protocol very interesting, and the clinical value of this intervention is high as it targets the need for rehabilitation support in unsupervised home-based physical rehabilitation as well as a technical solution for monitoring progress.
Response: *We thank the reviewer for taking time to review the manuscript and provide their valuable suggestions which we think has significantly improved the clarity of the manuscript.*

2. My biggest concern is that the intervention includes so many different features in the app which might be overwhelming for the study population to be included in this study. Has the app been developed and together with patients and caregivers? If so, was the usability of the app evaluated by the patients? I miss more information and discussion about this.
Response: *Thank you for raising this query. The intervention components were identified following a needs assessment conducted through literature reviews and surveys with patients and healthcare providers to identify challenges during post knee arthroplasty rehabilitation. Based on this data and upon discussion with orthopaedic surgeons, physiotherapists and behavioural experts, the intervention components (education, training, goal setting and self-management, communication, and access to personalized therapy) to improve adherence to exercise protocol during the early phase of rehabilitation (target behaviour) were finalised. The app components reflect these intervention functions.*
The comprehensibility of the education material, exercise videos, aesthetics, and navigation of the app was assessed from 10 volunteering patients using the app prototype. Following this, the app was pilot tested over a period of 4 weeks amongst individuals who had undergone knee arthroplasty (n=30) and their caregivers to assess the extent of use and the barriers and facilitators. We found that usability of the application was higher when either the patient or the caregiver was digitally literate. In this trial, among digitally literate participants we will be assessing the extent of use of different components and the reasons for usage and non-usage. We have now added a brief context of how the intervention was designed and tested prior to this trial in the introduction and intervention section as below.
Revision: *(Page 5, lines 1-3) "In response to this need for supervised home-based physical rehabilitation and a lack of available tools to support rehabilitation, we developed a multicomponent application [TeleREhabilitation after knee ArThroplasty (TReAT app)] to provide knowledge about recovery process, aid adherence to the rehabilitation therapy, and to facilitate remote therapy planning (9). The intervention functions to address the challenges in rehabilitation were finalized using behavior design thinking approach in consultation with orthopedic surgeons, physiotherapists and behavioral experts (9)."*
(Page 8, lines 29-33) "The comprehensibility of the education material, exercise videos, aesthetics and navigation of the app was assessed from 10 volunteering participants using the app prototype. The intervention was pilot tested on 30 participants and demonstrated high acceptability among users with digital literacy. Feedback from end users led to refinements in user interface, flexible scheduling of video consultations and addition of frequently asked questions and app manual."

3. Another overall comment is whether this study investigate implementation in real world scenarios or just in a research context, see specific comment under methods.
Response: *The study as it has been designed and now been implemented will give results of implementation in "research context" due to the involvement and influence of research coordinator in implementation of the intervention. In the discussion section, we have discussed about the level of pragmatism of this trial using the PRECIS-2 tool (Figure 3).*

4. ABSTRACT: To get a better understanding of the methods I would like a small description of what usual care means in this study.
Response: *Thank you for pointing this out. In India, the usual care after knee arthroplasty varies by*

hospital. Usually, it consists of written or oral instructions about wound care, do's and don't's from the surgeon and physiotherapist at the hospital before discharge. This is followed by a hospital visit to surgeon for suture removal, with or without a visit to the physiotherapist. Post this, follow ups occur as per individual need or surgeon discretion. The patients may hire home-based private physiotherapist or visit a nearby physiotherapy clinic or perform exercises on their own. We have now added a description of usual care in the abstract as below.
Revision: *(Page 2, lines 14-16) "..Consenting individuals will be randomized to either an app-supported intervention or a usual home-based rehabilitation which typically consists of provision of oral or written instructions at discharge and follow-up check-up with the surgeon or physiotherapist at their discretion or as per individual need."*

5. INTRODUCTION: Page 4, lines 20-21: It would help the reader if you included a definition of what internet-based telerehabilitation is.
Response: *Internet based telerehabilitation refers to delivering rehabilitation services via information and communication technologies that are internet-based, such as videoconferencing. We have now added the definition of internet based telerehabilitation in the text.*
Revision: *(Page 4 lines 8-9)" The effectiveness of various methods of internet-based rehabilitation monitoring (i.e. technologies that use internet for sharing information and communication), on knee pain and function, range of motion, satisfaction, and time to return to routine activities have shown mixed results (3)."*

6. Page 4, lines 56-57: Could you describe this study more, the results as well as elaborate a bit more about what changes you did after the pilot testing.
Response: *The objective of pilot testing was to check the usability of the intervention, gather insights about implementation of the intervention and guide further refinements. Overall, the patients and their family members valued the app-based rehabilitation support. The physiotherapists highlighted the importance of end user digital literacy to make users more compliant in using the app and in completing the diary. Post the pilot, the application was updated to include more user-friendly app manual, changes in the therapy planning and video consultation workflow, in-app video calling, a feature for additional resources and the availability of the application for iOS. Additionally, given the importance of digital literacy and in lieu of these findings we added digital literacy as an eligibility criterion for the trial. We have updated the text to reflect this as below.*
Revision: *(Page 5, lines 1-3) "In response to this need for supervised home-based physical rehabilitation and a lack of available tools to support rehabilitation, we developed a multicomponent application [TeleREhabilitation after knee ArThroplasty (TReAT app)] to provide knowledge about recovery process, aid adherence to the rehabilitation therapy, and to facilitate remote therapy planning (9). The intervention functions to address the challenges in rehabilitation were finalized using behavior design thinking approach in consultation with orthopedic surgeons, physiotherapists and behavioral experts (9)."*
(Page 8, lines 29-33) "The comprehensibility of the education material, exercise videos, aesthetics and navigation of the app was assessed from 10 volunteering participants using the app prototype. The intervention was pilot tested on 30 participants and demonstrated high acceptability among users with digital literacy. Feedback from end users led to refinements in user interface, flexible scheduling of video consultations and addition of frequently asked questions and app manual."

7. Page 4, lines 30-40: There are other programs around the world with different digital management programs for hip and knee osteoarthritis (OA) for example. I am missing more results and rationale around why digital/telerehabilitation can be a preferable solution for some patients. See for example a study by Cronström et al: "I would never have done it if it hadn't been digital': a qualitative study on patients' experiences of a digital management programme for hip and knee osteoarthritis in Sweden"

Response: *Thank you for this suggestion. In India, as per our previous research published here (7,8) we recognised that visiting hospital for follow-up or clinic-based monitoring was challenging due to logistic and economic reasons from the patients end. Having a physiotherapist visit at home did solve the commute problem for those who could afford. To ensure equitable quality continuum of care and given the widespread use of smartphones in India, remote tele based rehabilitation monitoring, which is well established in developed country context, seemed to be an option that required evaluation. We have now elaborated on the rationale in the background section.*

Revision*: (Page 4 lines 23-30) "..The usual rehabilitation care in India is mostly home-based with various levels of in-person supervision either at home or in clinic. Previous observational studies reported by our research group indicated a need for rehabilitation support to minimize out of pocket expenditure incurred due to home visits made by physiotherapist or clinic visits made by the patients (7). Specifically, patients found travel to hospitals and navigating within the hospital challenging, leading to poor adherence to rehabilitations protocols or receiving physiotherapy assistance from non-professional individuals (7, 8). Moreover, health care providers also expressed a need for a technology that could help them to monitor progress with functional recovery and be able to modify the therapy plan (9)."*

8. I am missing some background around the frameworks you are using for complex interventions, the framework by Linnan and Stecker. I think it would help understand the reasoning of your study to include a small section on this in the introduction and not only in the objectives.

Response: *Thank you for the suggestion. We have updated the introduction by adding a rationale for conducting the process evaluation.*

Revision: *(Page 5, lines 11-14) "Further, to understand if the proposed intervention leads to the expected behavioral change and subsequent changes in clinical outcomes, we will embed a process evaluation within this trial. This will also help us answer questions regarding generalizability and scalability of the intervention in varying contexts."*

9. Objectives: Be consistent in what you call the control condition. In the Primary objectives you call it "usual home-based rehabilitation" and in the secondary objective "usual care"

Response: *Thank you for pointing this out. We have now updated the text and used "usual home-based rehabilitation" to refer to the control group consistently throughout the manuscript.*

10. METHODS: Trial design: The intervention is planned to be open-labelled. I understand that it is difficult to blind the interventions but are there any things you could do to increase the possibility of blinding the participants, for example be vaguer in the information letter to participants so that they do not know what the two options are? I would like some discussion about this.

Response: *This is a very useful suggestion. However, we have described the test intervention and control (usual home-based rehabilitation) in the participant information sheet, and we could not blind the participants at the level of consent. We agree that this would lead to performance and outcome*

assessment bias contributed by the trial participants. To minimise bias from the measurer perspective we have an independent blinded assessor who will not be involved in recruitment to conduct the follow-up assessments. Further we believe the addition of performance-based measures assessed by the blinded assessor will minimise bias. We have added this as a limitation in the manuscript.
Revision: *(Page 3, lines 9&10) "The participants are aware of the group allocation; however, the outcomes are assessed by a blinded assessor."*

11. Trial design: again about the consistency in what you call the control condition, here you call it "usual care home-based rehabilitation", see also comment under objectives. My suggestion is that you choose one way of reporting this and use it throughout the manuscript.
Response: *Thank you for pointing this out. We have now updated the text and used "usual home-based rehabilitation" to refer to the control group consistently throughout the manuscript.*

12. Exclusion criteria 2: "Individuals unable to make a hospital visit at 3 and 6 months after surgery". Could you clarify the rationale behind this exclusion criterion? I'm concerned that excluding individuals who, for example, live far away or face economic barriers to participation may reduce the generalizability of the study findings. Couldn't they be included for some parts of the study, for example PROMs?
Response: *Given the open label nature of the trial, we wanted to give equal importance to patient reported outcome measures as well as performance-based assessments. We understand that excluding individuals unable to come at 3 and 6 months might affect generalisability, but we wanted to ensure that we collect both assessments. Based on our current screening log, only 10% participants have been excluded based on this criterion.*

13. Page 7, line 15: "Control intervention". See comments under objectives and methods about what to call the control intervention, be consistent with terminology to make it easier for the reader.
Page 7, line 32: "Test intervention", I suggest adding app-based rehabilitation since that is what you refer to in the objectives.
Response: *We have now updated the text and used "usual home-based rehabilitation" to refer to the control group and "app-based rehabilitation" to refer to intervention group consistently throughout the manuscript.*

14. Could you clarify how the physical therapists are recruited. This is unclear to me, and how many physical therapists there are for the app-based rehabilitation to give support via the app and create the therapy planning.
Response: *There are two types of physical therapists involved in this study. Clinical physiotherapists appointed by the hospital who provide physiotherapy care to those undergoing knee arthroplasty. The trial team will have no role in their recruitment or work routines. Only this clinical physiotherapist will manage the care of the patient, update therapy plans and conduct video consultations. The trial team has appointed a research coordinator with a physiotherapy background who is trained in research conduct and familiar with post knee arthroplasty rehabilitation protocol. They will assist the clinical physiotherapist to enrol the patient into the app, remind about the video consultations and resolve any app navigation related queries for the end users. At present, there are three physiotherapists from the two hospital sites involved in the trial and two appointed physiotherapists to coordinate the trial related activities.*

15. Page 10, lines 50-60: A research coordinator will be available for technical help with the app both on-site in the beginning as well as doing check-in phone calls to assist with the app. My concern regarding this approach is that you evaluate whether the research project works with all this support and then you do not know if the intervention works later when the support is not there anymore when the project stops. Have you thought about this and maybe not providing this service, hence only see how the intervention works without this support? We know from, for example, prevention studies that injuries decrease when the physical therapists are in teams helping during studies with the prevention training but when the research stops the injuries increase again. So, the most important question is how do we implement this so that it works in the real world?
Response: *We totally agree with the reviewers' concerns. This trial would answer if the approach of remote monitoring is feasible and will have an impact on recovery. However, the process evaluation will reveal the intervention fidelity and the contextual challenges. This may help to address the operational question of how to implement such an intervention in future mHealth studies. This has been discussed in the discussion section (Page 19, lines 12-24).*

16. Page 11: "Blinding", please clarify if the research coordinator performing the data collection of the outcomes will be blinded to the interventions, this should be possible and are highly recommended.
Response: *The research coordinator performing data collection will be blinded to intervention group assignment and will also not be involved in the recruitment and rehabilitation care of the patient. We have revised the text to reflect this.*
Revision: *(Page 12, line 2) "This will be an open-label trial owing to the nature of the intervention. The outcome will be collected by a research coordinator who will be blinded to intervention assignment and will not be involved in recruitment and rehabilitation care of the participant."*

17. Page 11: "Outcomes", Why are you planning to distribute the KOOS questionnaire on paper and not through for example REDCap? By using REDCap you could include patients who are not able to come to the follow-up visits but still be able to answer questionnaires online.
Response: *We agree that using REDCap to complete KOOS questionnaire would be ideal. However, based on our prior experiences of conducting studies in tertiary care hospitals, using a web-based data entry interface is risky due to patchy internet. Moreover, in a LMIC context, the hospitals are overcrowded with paucity of space and a separate room for research is mostly not available. Thus, paper-based data entry is a more feasible and pragmatic in this setting.*

18. Page 12: Secondary outcomes, there is a need for a more detailed description of the physical tests, as it is now it is not possible to reproduce the study method without going into each of the reported references, which is inconvenient for the reader.
Response: *Thank you for the suggestion. We had developed a simple illustrative instruction manual for conducting these tests. We have now added this instruction manual (eFigure 8) as a supplementary information to the manuscript.*

19. Page 12: Please write out the explanation of the different secondary outcomes in the text and not only under Table 2. It makes it difficult to read when it is under the table. And, please provide them with appropriate references for the outcomes when those are missing.

Response: *Thank you for this suggestion. We have modified the text to include details of all outcomes as below.*
Revision: *(Page 12, lines 18-31) "Secondary outcomes: Other quantitative outcomes include pain, assessed using the 9-item KOOS-Pain questionnaire (15) with a 5-point ordinal response scale, higher scores indicating higher levels of pain. Activity level will be assessed using the lower extremity activity scale (17) a single item self-reported question with 18 states of activity, higher numbers indicating higher level of activity. Quality of life will be assessed using EQ-5D-5L questionnaire (18), capturing 5 domains of health namely mobility, self-care, usual activities, pain/discomfort, and anxiety/depression. The overall score ranges from 0-1, higher score indicating better quality of life. Safety, knowledge about recovery, rehabilitation adherence, satisfaction with surgical outcome, and cost incurred will be collected using study specific questionnaires. Active and passive knee range of motion (19) of the operated knee(s) will be measured using a digital goniometer. Performance-based measurements include chair rising test to assess lower limb strength, stair climbing and 6-minute walk tests to assess knee function, timed-up and go to assess gait stability (20), and single leg stance to assess balance (21). Further explanation of the measurement tools is found in Table 2 and the standardized procedure to conduct the performance-based tests is illustrated in eFigure 8 in supplementary file."*

20. Page 13, "Process outcomes" lines 23-27. It is difficult to understand how you are going to measure self-efficacy to rehabilitation, this needs more description.
Response: *The ability to adhere to the rehabilitation protocol is measured using a 22-item questionnaire developed by us to capture the capability, opportunity and motivation of an individual. This would indirectly capture self-efficacy for rehabilitation. This questionnaire was developed based on the rehabilitation adherence questions designed for sports related knee injury reported by Shin and Park (22). However, our questionnaire is not validated for all the psychometric properties.*
Revision*: (Page 14, lines 8-12) "To gain understanding of the mechanistic pathway to change, we will measure self-efficacy to rehabilitation. This questionnaire was developed based on the rehabilitation adherence questions designed for sports related knee injury by Shin and Park (22). However, our questionnaire is not validated for all the psychometric properties. We will also record knowledge on recovery after surgery using a set of knowledge questions and collect the list of exercises performed from both the groups."*

21. Page 13: Economic component. Please specify if that this is in both groups.
Response: *Yes, we are collecting cost incurred during the six-month period after surgery from both the groups. Using this information, we will be able to compare out-of-pocket expenditure incurred by the two groups. We hypothesis that due to the availability of mHealth application, the out-of-pocket expenses will be reduced in the intervention group when compared to the usual care group. If the intervention is proven to be effective (better knee function score), we will undertake a cost utility analysis using quality of life at six-months as the outcome. We have revised the text to reflect this.*
Revision: *(Page 14, line 15)* ***"….*** *Resource use from patient perspective that includes cost of physiotherapy services, travel expenditures and wages lost due to clinic visits will be measured using study specific questionnaires at 3 and 6 months in both groups…"*
(Page 17, line 16) "Cost analysis: Costing components will be considered from the patients' perspective in both groups."

22. Page 14, lines 2-13: Why are you using this approach and not collecting the data online directly via REDCap? Page 14, lines 57-60: Again, if using the online REDCap this problem is solved.
Response: *Based on our prior experiences of conducting studies in tertiary care hospitals, using a web-based data entry interface is risky due to patchy internet services. Moreover, in the LMIC context, there is overcrowding, paucity of space and a separate room for research is mostly not available. Thus, paper-based data entry is a more feasible and pragmatic in this setting.*

23. Page 15, Public involvement: I think this is important information that you have had patients testing the app before this trail and I missed this information earlier in the manuscript. I think it would be nice if you could add more description of this and results from this study on page 7 "Test intervention" to clarify this earlier for the reader.
Response: *Thank you for this suggestion. We have now added this information in the description of the intervention.*
Revision: *(Page 8, lines 29-33) "The comprehensibility of the education material, exercise videos, aesthetics and navigation of the app was assessed from 10 volunteering participants using the app prototype. The intervention was pilot tested on 30 participants and demonstrated high acceptability among users with digital literacy. Feedback from end users led to refinements in user interface, flexible scheduling of video consultations and addition of frequently asked questions and app manual."*

VERSION 2 - REVIEW

**Reviewer**	**2**
**Name**	**Nae, Jenny**
**Affiliation**	**Lund University, Department of Health Sciences**
**Date**	**21-Oct-2025**
**COI**	

I congrat the authors to a well written study protocol and an interesting and important study. I am pleased with the answerers and changes made to manuscript based on my comments. I have no further points to raise.
